# The Strehler-Mildvan mortality correlation arises from changes in the variability of ageing

**DOI:** 10.64898/2026.02.15.705972

**Published:** 2026-02-17

**Authors:** Bruce Zhang, Yiran Zhang, Zibo Gong, David Gems

**Affiliations:** Institute of Healthy Ageing, and Research Department of Genetics, Evolution and Environment, University College London, London, UK

**Keywords:** Ageing, *C. elegans*, gerospan, Gompertz equation, healthspan, lifespan, mortality rate, Strehler-Mildvan correlation

## Abstract

As global human life expectancy continues to rise, accompanying increases in healthspan that prevent morbidity expansion become increasingly imperative. Population lifespan can increase in distinct ways, for instance through rectangularisation (steepening) or triangularisation (flattening) of survival curves. These two demographic changes, particularly rectangularisation, occur frequently across human and model organism populations, yet their biological determinants and effects on healthspan and morbidity are largely unknown. Notably, these modes of life-extension occur when parameters of the Gompertz mortality model (capturing exponential age-increases in mortality rate) change inversely, a widely-reported phenomenon known as the Strehler-Mildvan correlation – whose biological basis also remains unexplained. We therefore investigated longitudinal health, morbidity and lifespan in 30 *Caenorhabditis elegans* cohorts using multiple life-extension protocols. We report that survival curve rectangularisation results from healthspan expansion in short-lived population members, whereas triangularisation from healthspan and morbidity expansion in long-lived population members. Interestingly, rectangularisation and triangularisation respectively decrease and increase inter-individual variation in the ageing process, and the mode of life-extension that occurs depends on levels of existing variation. Notably, triangularisation was more effective at extending lifespan without morbidity expansion. Analysis of fruit fly and mouse data show that these biological determinants of the Strehler-Mildvan correlation are also largely evolutionarily conserved.

## Introduction

The biological process of ageing (senescence) is a leading cause of mortality and morbidity worldwide (Guo et al., 2022), yet its causes and mechanisms remain poorly understood. Given continued increases in global life expectancy (GBD 2021 Forecasting Collaborators, 2024), concomitant changes in the durations of health (healthspan) and morbidity, and their biological determinants, become increasingly important to quantify and understand.

**Fig. 1.**
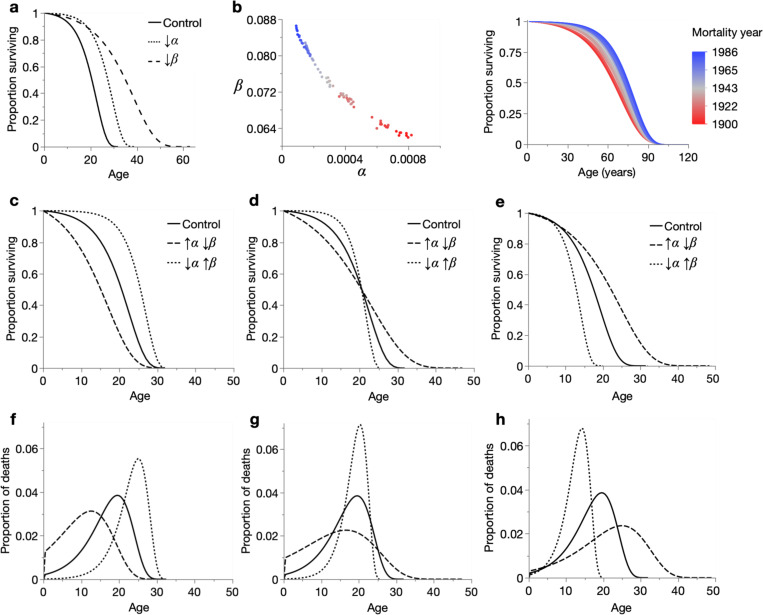
The Strehler-Mildvan correlation and its demographic effects. (**a**) Effect of decreasing Gompertz parameters α and β on the survival curve. **Control**: α, β: 0.002, 0.2; ↓α: α, β: 0.0005, 0.2; ↓β: α, β: 0.002, 0.1. (**b**) Left: inverse relationship between the Gompertz parameters from annual period mortality (i.e. all deaths that year). Right: equivalent Gompertz survival curves, of U.S. men between 1900 and 1986, produced from data in Riggs (1990). (**c**–**e**) Three forms of survival curve transformation characteristic of the S-M correlation: (**c**) rectangularisation/de-rectangularisation, (**d**) intersection of survival curves, and (**e**) triangularisation/de-triangularisation. In these examples, α and β have been set so that maximum (**c**), median (**d**) and minimum (**e**) lifespans are approximately equivalent, but these values can vary. The parameters are: (**c**) **Control**: α=0.002, β=0.2; ↑α↓β: α=0.012, β=0.14; ↓α↑β: α=0.0001, β=0.29, (**d**) **Control**: α=0.002, β=0.2; ↑α↓β: α=0.012, β=0.08; ↓α↑β: α=0.0001, β=0.37, and (**e**) **Control**: α=0.002, β=0.2; ↑α↓β: α=0.003, β=0.12; ↓α↑β: α=0.0012, β=0.35. (**f**–**h**) Respectively, effects of Gompertz parameter changes in **c**–**e** on mortality frequency over time.

Lifespan increases can be studied through mortality modelling, such as with the Gompertz equation (Gompertz, 1825), $\mu (x)=\alpha e^{\beta x}$, which can capture the exponential increase in mortality rate observed in many animal species, including humans. Here, mortality rate at age x is determined by a scale parameter α and rate parameter β, which respectively specify the theoretical initial mortality rate (at x=0) and mortality rate acceleration with advancing age. These parameters have different effects on the survival curve (Fig. 1a). Although the Gompertz model is frequently employed in ageing research, the biological meaning of α and β has only recently been demonstrated empirically (Zhang and Gems, 2026).

A further enigma is the Strehler-Mildvan (S-M) correlation: an inverse correlation between α and β frequently observed between human populations (Strehler and Mildvan, 1960, Riggs, 1990, Prieto et al., 1996), most notably in the steepening of global survival curves over the last two centuries (Fig. 1b). The correlation has since also been reported between laboratory animal populations (Krementsova et al., 2012, Zhikrevetskaya et al., 2015, Shen et al., 2017, Golubev et al., 2018). In a modified form, the S-M correlation is also known as the compensation law of mortality (Gavrilov and Gavrilova, 1991).

The S-M correlation (i.e. any inverse change in α and β) has distinctive effects upon the survival curve, for instance causing rectangularisation/de-rectangularisation (Fig. 1c, 1b right), intersection (crossing over) (Fig. 1d), or triangularisation/de-triangularisation (Fig. 1e). The latter term (triangularisation) we introduce in this study. Lifespan extension can therefore occur through decreased α and increased β (rectangularisation) or through increased α and decreased β (triangularisation); in the life-shortening direction, these changes become de-rectangularisation and de-triangularisation, respectively. Importantly, because the Gompertz parameters affect lifespan in the same direction (decreasing α or β both increase lifespan; Fig. 1a), S-M correlations always involve antagonistic lifespan effects. Thus, depending on the magnitudes of inverse α and β change, survival curves can converge anywhere along their lengths: at late ages in rectangularisation (Fig. 1c), early ages in triangularisation (Fig. 1e), or somewhere in between in the case of intersection (Fig. 1d). These S-M correlations have intuitive effects upon the mortality frequency distribution, which is approximately shifted and stretched/compressed by α and β, respectively (Fig. 1f–h).

However, the biological basis of the S-M correlation, in terms of changes in the ageing process, remains unclear and has not been determined empirically. Possible explanations include the effects of antagonistic pleiotropy (Yashin et al., 2001) and intra-population heterogeneity (Vaupel and Yashin, 1985, Yashin et al., 2001, Hawkes et al., 2012). S-M correlations may also arise from modelling artefacts (Lestienne, 1988, Burger and Missov, 2016, Tarkhov et al., 2017) but, as we will show, not in the data we present here. Importantly, effects of the S-M correlation on durations of healthspan and morbidity have not been directly studied.

We recently investigated the biological basis of the Gompertz parameters in 24 differently-lived cohorts of *C. elegans*. Our findings showed that, contrary to long-standing hypothetical interpretations, α and β respectively, reflect biological ageing rate and inter-individual variability in morbidity duration (Zhang and Gems, 2026). In the present study, we revisit and add to these earlier cohorts to examine empirically the biological changes underpinning the S-M correlation. We find that rectangularising S-M correlations (survival curve rectangularisation) reflect healthspan expansion in shorter-lived population members and increased homogeneity in the ageing process. In contrast, triangularising S-M correlations (survival curve triangularisation) reflect both healthspan and gerospan (morbidity) expansion in longer-lived population members and increased heterogeneity in the ageing process. Notably, similar biodemographic dynamics are seen in fruit fly and mouse populations exhibiting the S-M correlation. Our findings offer an empirical, biological explanation of the Strehler-Mildvan correlation (see Fig. 9 for a schematic overview), whose determinants may be conserved between invertebrates and mammals.

## Results

### Lifespan-extending treatments in *C. elegans* produce S-M correlations

Despite frequent use of *C. elegans* lifespan data in Gompertzian mortality modelling, the existence and biological significance of S-M correlations in such data have been little studied. To address this, we produced a longitudinal health and mortality dataset of 30 nematode cohorts, comprised of all combinations of 3 culture temperatures (15°C, 20°C, 25°C), with/without antibiotic (carbenicillin), and 5 genotypes (wild type, *daf-2(m577)*, *daf-2(e1368)*, *daf-2(e1370)*, *daf-16(mgDf50)*). We recently used 24 of these cohorts to investigate the biological basis of the Gompertz parameters (Zhang and Gems, 2026), and here present the completed dataset that also includes 6 *daf-16* cohorts (at 3 temperatures, with/without carbenicillin), additional necropsy data (of end-of-life pathology), and measurements of early-adulthood bacterial lawn (food) interactions for each cohort.

Gompertz parameters were obtained from the lifespan data of each cohort by maximum likelihood estimation (Pletcher, 1999) (Supplementary Table 1). These Gompertz fits were verified with parametric Anderson-Darling goodness-of-fit tests (Supplementary Table 2), and additionally predicted first quartile, median and third quartile lifespans with R^2^ of 0.981, 0.996, 0.993, respectively (Extended Data Fig. 1). Each cohort (mean *n*=144 individuals/cohort) was pooled from at least 3 sequential trials, whose inter-trial lifespan variation was low (Extended Data Fig. 2, Supplementary Table 1). We also previously showed that increasing population sizes for 24 of these cohorts by ~3-fold (>300 individuals/cohort) had negligible effects on lifespan and Gompertz parameter estimates (Zhang and Gems, 2026).

The 30 cohorts yield in total 435 pairwise comparisons between them, each reflecting a distinct biological change in the ageing process and lifespan. We therefore refer to these 435 comparisons as *treatments* and evaluate them in the lifespan-extending direction (i.e. 435 lifespan-extending treatments). This pairwise approach not only informs about lifespan extension by specific conditions/mechanisms (temperature, antibiotics, IIS pathway mutations, and their interactions) but makes possible the study of condition-independent patterns, i.e. fundamental principles that govern the relationship between demographic and biological ageing.

**Fig. 2.**
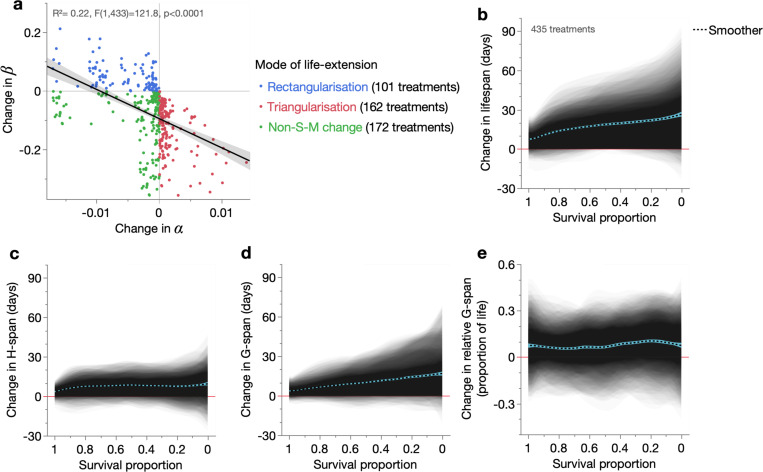
Modes of lifespan extension and effects on healthspan and gerospan. (**a**) Least-squares linear regression of changes in β between all possible pairs of the 30 cohorts, over the corresponding change in α for those pairs. Change direction was always determined based on the longer-lived cohort minus shorter-lived cohort, such that all changes reflect the effects of lifespan extension. The relationship was assessed by an F-test and the 95% confidence region shaded. (**b**–**e**) Shaded and overlaid (JMP transparency=0.02) area plots for all 435 treatments (comparison pairs), of the change in (**b**) lifespan, (**c**) H-span, (**d**) G-span, and (**e**) relative G-span. These shaded changes are plotted over survival proportion (x-axis left: shorter-lived population members, x-axis right: longer-lived population members). Red line: y=0, below which the lifespan-extending treatment shortens that trait. Dashed black line: summary spline smoother (lambda=0.05) of the 435 treatment changes, showing its 95% confidence region in blue.

We began by assessing the presence and type of S-M correlations in the dataset. An inverse relationship was observed between changes in α and β across the 435 lifespan-extending treatments, revealing an S-M correlation across all conditions (Fig. 2a). 169/435 (39%) of treatments increased lifespan by decreasing both Gompertz parameters (i.e. non-S-M treatments), while 263/435 (60%) increased lifespan through inverse parameter changes: 101 treatments decreased α and increased β (i.e. rectangularisation), and 162 increased α and decreased β (i.e. triangularisation). Thus, lifespan extension amongst these 30 cohorts arises predominantly through S-M correlations: rectangularising S-M correlations that steepen the survival curve (Fig. 1c), and triangularising S-M correlations that flatten it (Fig. 1e). Here, our definition of S-M correlation includes inverse Gompertz parameter changes between just two cohorts, which we will refer to as rectangularising or triangularising S-M treatments (S-M^rect^ and S-M^tri^ treatments, respectively).

As expected, no treatments increased both Gompertz parameters (which would decrease lifespan) (Fig. 2a), reaffirming that the estimated parameters accurately capture lifespan changes in these treatments. This, and the robust fitting of the Gompertz model to these cohorts (Extended Data Fig. 1, Supplementary Table 2) show that the particular S-M correlations within this dataset are not statistical artefacts, which can arise under certain conditions (Lestienne, 1988, Burger and Missov, 2016, Tarkhov et al., 2017). Here, our S-M treatments and their S-M correlations reflect real (biological) changes in lifespan and thus also the ageing process. Furthermore, exclusion of treatments in which one or both Gompertz parameters were not statistically significantly changed even strengthened the overall treatment-wide S-M correlation, and predominantly excluded non-S-M rather than S-M treatments (Extended Data Fig. 3).

### Different dynamics of health and morbidity during lifespan extension

We then addressed the focus of this study: the biological features of the ageing process that generate S-M correlations. To this end, alongside collecting lifespan data, we longitudinally tracked the health of each individual nematode from reproductive maturity until death, in all 30 cohorts. This included assessment of locomotory capacity every 2–3 days, interaction with the *E. coli* food source every 2–3 days, and *E. coli* infection status at the time of death. Such simultaneous quantification of biological ageing traits and lifespan in the same individuals can reveal the biological basis of demographic mortality patterns (Zhang and Gems, 2026, Zhao et al., 2017), an approach we also employ here to investigate the biological determinants of the S-M correlation.

We first visualised the effects of the 435 treatments on lifespan, by shading the change in survival curves for each treatment, plotted over a survival proportion x-axis (left: shorter-lived population members, right: longer-lived population members) (Fig. 2b). This mode of presentation shows in which population members a change of interest (here, lifespan) occurs. As expected, the 435 treatments increased lifespan and, as often observed in nematode survival studies (Stroustrup et al., 2016, Zhang et al., 2016, Zhang and Gems, 2026), more so in longer-lived population members (Fig. 2b). Lifespan of the shortest and longest-lived population members was also modestly decreased in a few cases, indicating antagonistic lifespan effects (i.e. intersecting survival curves).

We similarly examined effects of these treatments on ageing-related health and morbidity, here respectively measured as youthful (sinusoidal) and reduced (non-sinusoidal or immotile) locomotory capacity, which are physiologically-integrative measures of the ageing process (Hosono et al., 1980, Herndon et al., 2002, Zhang and Gems, 2026). We refer to the number of days of life spent in youthful and reduced locomotory capacity as *healthspan* (H-span) and *gerospan* (G-span), respectively, and the proportion of life spent in gerospan as *relative gerospan* (relative G-span). Because H-span + G-span = lifespan, we can explain changes in lifespan in terms of changes in H-span and G-span (i.e. a biological explanation of mortality patterns).

As expected given lifespan extension, both H-span and G-span were mainly increased across the 435 treatments (Fig. 2c–d). Notably, the magnitude of G-span increase was greater, attributable to a greater increase in longer-lived population members. Consistent with this was an overall increase in relative G-span (Fig. 2e). These results align with our earlier analysis of 46 treatments from this dataset (Zhang and Gems, 2026), in which (1) lifespan extension arose primarily from G-span expansion, expanding the proportion of life spent in decrepitude, and (2) G-span expansion was more inter-individually variable than H-span expansion, such that they better explained reductions in, respectively, β and α. These relationships between overall health, morbidity and lifespan could potentially represent basic biodemographic principles, that are to some degree independent of specific treatments and ageing mechanisms.

### Lifespan rectangularisation and triangularisation reflect distinct changes in health and morbidity

**Fig. 3.**
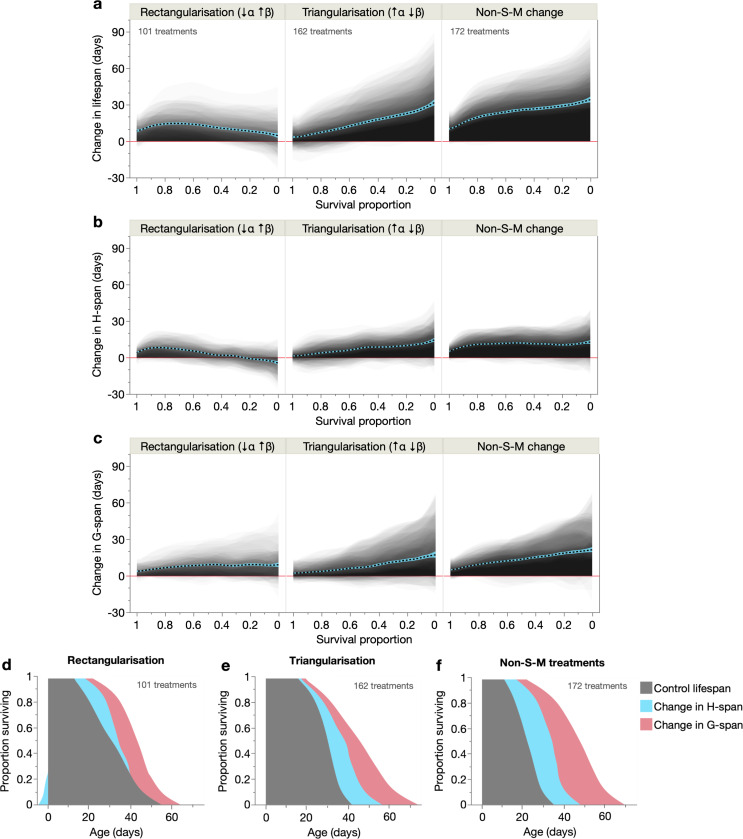
Different demographic lifespan extension modes have distinct effects on healthspan and gerospan. (**a**–**c**) Shaded and overlaid (JMP transparency=0.02) area plots for all treatments (comparison pairs) of each demographic treatment type (S-M^rect^, S-M^tri^, non-S-M), of the change in (**a**) lifespan, (**b**) H-span, and (**c**) G-span. These shaded changes are plotted over survival proportion (x-axis left: shorter-lived population members, x-axis right: longer-lived population members). Red line: y=0, below which the lifespan-extending treatment shortens that trait. Dashed black line: summary spline smoother (lambda=0.05) of the 435 treatment changes, showing its 95% confidence region in blue. The number of treatments in each demographic type is annotated in **a**. (**d**–**f**) Empirical summary figures of how S-M^rect^, S-M^tri^ and non-S-M treatments extend lifespan through additive changes in H-span and G-span (spline smoother of each, stacked on spline smoother of control lifespan), displayed in a shaded survival curve-like format. Shortening of H-span in the longest-lived population members in S-M^rect^ treatments is represented as a negative change relative to Age=0; this has the effect of further steepening the resultant survival curve.

We then asked if these changes in H-span and G-span depend on the mode of lifespan extension: from S-M^rect^, S-M^tri^ or non-S-M treatments. First, we examined effects of these three treatment types on lifespan (Fig. 3a); as expected, S-M^rect^ treatments increased lifespan more in short-lived population members (steepening the survival curve), S-M^tri^ treatments in long-lived population members (flattening the survival curve), and non-S-M treatments produced a mix of these lifespan changes. Interestingly, lifespan shortening by some treatments at the highest and lowest survival proportions (Fig. 2b) is here revealed to reflect lifespan shortening in the longest-lived population members in S-M^rect^ treatments, and shortest-lived population members in S-M^tri^ treatments (Fig. 3a).

Mirroring these lifespan changes, H-span was increased by S-M^rect^ treatments in shorter-lived population members, by S-M^tri^ treatments in longer-lived population members, and by non-S-M treatments relatively evenly across survival proportions (Fig. 3b). Interestingly, H-span was even decreased in the longest-lived population members, explaining their modest lifespan shortening (Fig. 3a). Meanwhile, all three modes of lifespan extension increased G-span primarily in longer-lived population members, moderately in S-M^rect^ treatments and strongly in S-M^tri^ and non-S-M treatments (Fig. 3c). These results reveal distinct combinations of change in H-span and G-span for each mode of lifespan extension, reflecting distinct patterns of change in the biological ageing trajectory. Given that each population is isogenic and cultured under identical conditions, the reproducible inter-individual variability of H-span and G-span (e.g. selective extension in short- or long-lived individuals) is striking.

These findings provide a high-level biological view of demographic ageing across the 30 cohorts, as individually-resolved distributions of ageing-related health and morbidity. The two distributions of health and morbidity combine additively to yield the lifespan distribution, thus providing empirical, biological explanations for changes in survival curve shape. These findings are summarised in a shaded survival curve format (Fig. 3d–f). S-M^rect^ treatments extend H-span in shorter-lived population members (even truncating it in the longest-lived) and G-span more equally across the population (Fig. 3d). This shows that survival curve rectangularisation results directly and entirely from changes in H-span (not G-span), because its selective expansion in short-lived population members postpones early mortality, thus steepening the survival curve. Furthermore, modest H-span shortening in the longest-lived population members truncates the survival curve tail (i.e. maximum lifespan), thereby further steepening and rectangularising the curve.

In contrast, S-M^tri^ treatments extend both H-span and G-span more in longer-lived population members (Fig. 3e), such that both are responsible for the survival curve tail lengthening that occurs in triangularisation. Finally, non-S-M treatments extend H-span equally in all population members and G-span in longer-lived members (Fig. 3f). Here, H-span expansion effectively shifts the survival curve rightward while G-span expansion extends its tail, corroborating our recent findings (Zhang and Gems, 2026). Notably, these H-span and G-span changes resulting from the non-S-M treatments are intermediate between those of S-M^rect^ and S-M^tri^ treatments, consistent with lifespan extension in both short- and long-lived population members.

### Morbidity expansion and compression by lifespan rectangularisation and triangularisation

**Fig. 4.**
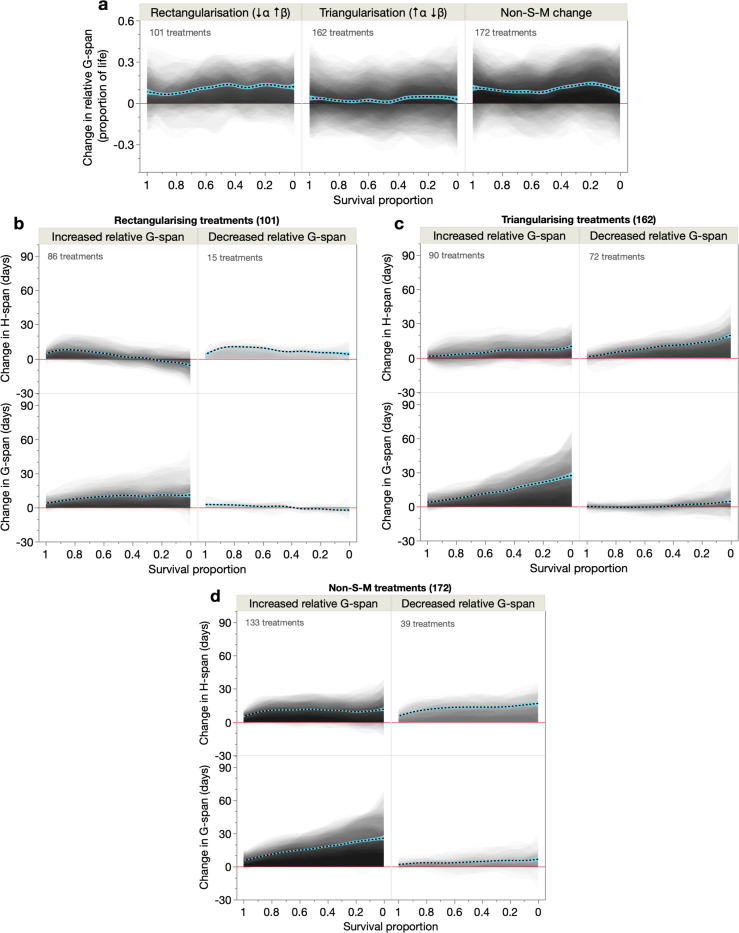
Effects of S-M and non-S-M treatments on morbidity expansion and compression. Shaded and overlaid (JMP transparency=0.02) area plots for all treatments (comparison pairs) of each demographic treatment type (S-M^rect^, S-M^tri^, non-S-M), of the change in (**a**) relative G-span, and (**b**–**d**) H-span and G-span for those subset treatments that increase or decrease relative G-span. These shaded changes are plotted over survival proportion (x-axis left: shorter-lived population members, x-axis right: longer-lived population members). Red line: y=0, below which the lifespan-extending treatment shortens that trait. Dashed black line: summary spline smoother (lambda=0.05) of the changes, showing its 95% confidence region in blue. The number of treatments in each category is specified.

We wondered how treatments affect the proportion of life spent in age-related morbidity. Notably, all three modes of lifespan extension increased relative G-span (proportion of life in G-span), strongly in S-M^rect^ and non-S-M treatments and moderately in S-M^tri^ treatments (Fig. 4a). Thus, lifespan extension across these 30 cohorts predominantly causes morbidity expansion rather than compression, and irrespective of the demographic mode of longevity. This is consistent with prior reports of morbidity expansion in long-lived *C. elegans* (Huang et al., 2004, Bansal et al., 2015, Podshivalova et al., 2017, Statzer et al., 2022, Zhang and Gems, 2026), although different morbidity measures in certain strains have also suggested proportional morbidity scaling (Hahm et al., 2015, Statzer et al., 2022).

However, some treatments, especially S-M^tri^ treatments, did decrease relative G-span (Fig. 4a). Such morbidity-compressing treatments are of particular interest; to study them further we partitioned treatments that expand versus compress morbidity, for each demographic treatment class (Fig. 4b–d). As expected, most treatments increased relative G-span, via similar demographic changes in H-span and G-span to that observed across all treatments (Fig. 3b–c) but with greater magnitudes of G-span increase.

Meanwhile, the number of S-M^rect^, S-M^tri^ and non-S-M treatments that compressed morbidity was, respectively, 15/101 (15%), 72/162 (44%) and 39/172 (23%) (Fig. 4b–d). Thus, most morbidity-compressing treatments (87/126: 69%) are S-M treatments (by comparison, 60% of all treatments are S-M treatments), and most of these morbidity-compressing S-M treatments (72/87: 83%) are S-M^tri^ treatments. Therefore, within this dataset, S-M^tri^ treatments are best able to compress morbidity while extending lifespan.

How does such morbidity compression arise, in terms of changes in H-span and G-span? The morbidity-compressing S-M^rect^ treatments increased H-span across all survival proportions, with greatest increases in short-lived population members, while G-span remained largely unchanged (Fig. 4b, right). Similarly, the morbidity-compressing S-M^tri^ treatments robustly increased H-span (in longer-lived population members) but not G-span (Fig. 4c, right). Likewise, the morbidity-compressing non-S-M treatments increased H-span markedly more than G-span (Fig. 4d, right). Compared to the morbidity-expanding treatments (left panels), these morbidity-compressing treatments increased H-span more, and strongly suppressed G-span increases. Thus, these treatments extend lifespan almost entirely through a large H-span extension, such that these individuals experience a postponed *and* brief period of morbidity (Extended Data Fig. 4a–c).

**Fig. 5.**
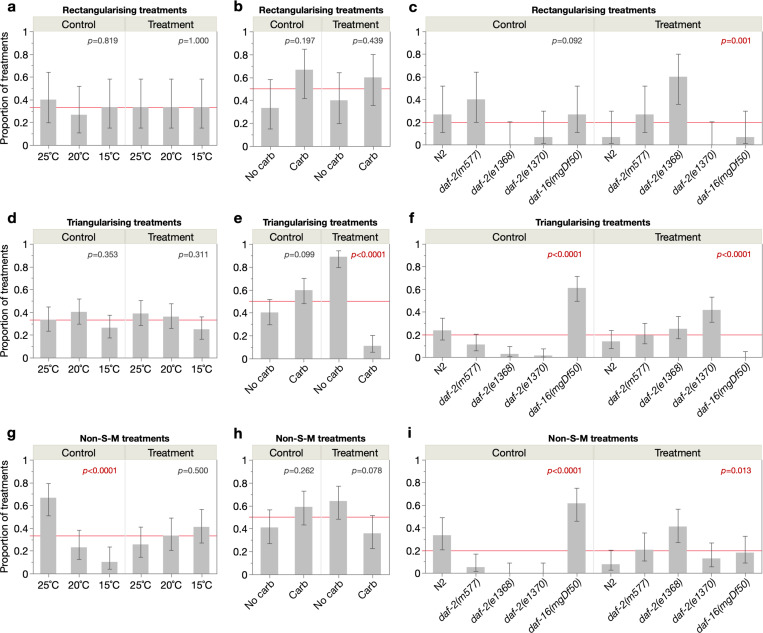
S-M and non-S-M treatments compress morbidity through shared and distinct mechanisms. Proportions of the control and treatment cohorts belonging to each experimental condition (3 culture temperatures, ± carbenicillin, 5 IIS-related genotypes), for treatments that decrease relative G-span (i.e. compress morbidity) through (**a**–**c**) S-M^rect^, (**d**–**f**) S-M^tri^ or (**g**–**i**) non-S-M demographic changes. Each lifespan-extending treatment comprises a control and treatment cohort, which respectively inform about the mechanistic background and intervention required to compress morbidity. Proportions within each control and treatment panel sum to 1, and proportions above or below the red lines (expected proportions under null hypothesis: 0.33 for 3 temperatures, 0.5 for ± carbenicillin, 0.2 for 5 genotypes) indicate enrichment or depletion of that condition. 95% confidence intervals are shown and Pearson chi-square goodness-of-fit tests were run for each panel (*p* values annotated), with sample sizes of, S-M^rect^: 15 treatments, S-M^tri^: 72 treatments, and non-S-M: 39 treatments.

An interesting question is whether morbidity-compressing treatments involve particular experimental conditions. We therefore asked whether specific temperatures, presence or absence of antibiotic, or specific genotypes were either more or less prevalent than expected by chance, in the control and treatment cohorts of each pairwise treatment class (S-M^rect^, S-M^tri^ and non-S-M) (Fig. 5, Supplementary Tables 3–5). Amongst the S-M^rect^ treatments, genotype, but not temperature or antibiotic usage, was associated with morbidity compression (Fig. 5a–c). Specifically, *daf-2(e1368)* was enriched amongst the treatment cohorts, revealing it to be a major cause of morbidity compression in these S-M^rect^ treatments. This suggests that unlike other stronger *daf-2* alleles (e.g. *daf-2(e1370)*), *daf-2(e1368)* both maintains wild type-like levels of voluntary locomotion (as known (Podshivalova et al., 2017, Statzer et al., 2022)) *and* exhibits prolonged true locomotory capacity (detected here through *stimulated* locomotion).

Amongst the S-M^tri^ treatments, both antibiotic usage and genotype were associated with morbidity compression (Fig. 5d–f). This was associated with the *absence* of antibiotic, likely reflecting the morbidity-unmasking effects of preventing life-limiting infection (Podshivalova et al., 2017, Zhang and Gems, 2026) (Fig. 5e). Meanwhile, being enriched amongst control cohorts (Fig. 5f), *daf-16(mgDf50)* was the most responsive genotype to triangularising morbidity compression (resulting primarily from *daf-2(e1370)* mutation), consistent with the view that mutation of *daf-16* accelerates ageing (Kenyon et al., 1993, Lin et al., 2001) (producing particularly short H-spans). Finally, amongst the non-S-M treatments, cohorts inhabiting higher temperatures (25°C) and again, *daf-16(mgDf50)* cohorts, were particularly responsive to morbidity compression (Fig. 5g–h).

Therefore, these S-M^rect^, S-M^tri^ and non-S-M treatments postpone morbidity through common and distinct mechanisms associated with, respectively, IIS, infection and IIS, and temperature and IIS. We also assessed the number of simultaneous condition changes (e.g. 1=temperature change only, 2=temperature and antibiotic usage changes, 3=temperature, antibiotic usage and genotype changes) required for these treatments to compress morbidity. Interestingly, in S-M^rect^ and S-M^tri^ treatments, 2 condition changes were most common (Extended Data Fig. 4d), suggesting that interactions between these conditions are required to postpone morbidity, rather than any one alone.

### The S-M correlation captures fluctuations in the inter-individual variability of ageing

Our longitudinal dataset is informative of not only the biological ageing trajectory but its inter-individual variability. An important goal of ageing research is to explain lifespan variation, both between and within population; the latter is particularly intriguing in isogenic and environmentally controlled populations, as in this dataset. In *C. elegans*, lifespan variation is particularly caused by inter-individual variation in the duration of late-life morbidity (Zhang et al., 2016, Churgin et al., 2017, Statzer et al., 2022, Zhang and Gems, 2026), and correlates with early life molecular and cellular traits (Baeriswyl et al., 2010, Shen et al., 2014, Tiku et al., 2017, Bazopoulou et al., 2019, Papsdorf et al., 2023). Therefore, we wondered whether the decreased and increased lifespan variation, respectively, in lifespan rectangularisation and triangularisation (S-M^rect^ and S-M^tri^ treatments) is determined by similar variability changes in the preceding biological ageing process (e.g. in H-span and G-span, amongst other traits).

**Fig. 6.**
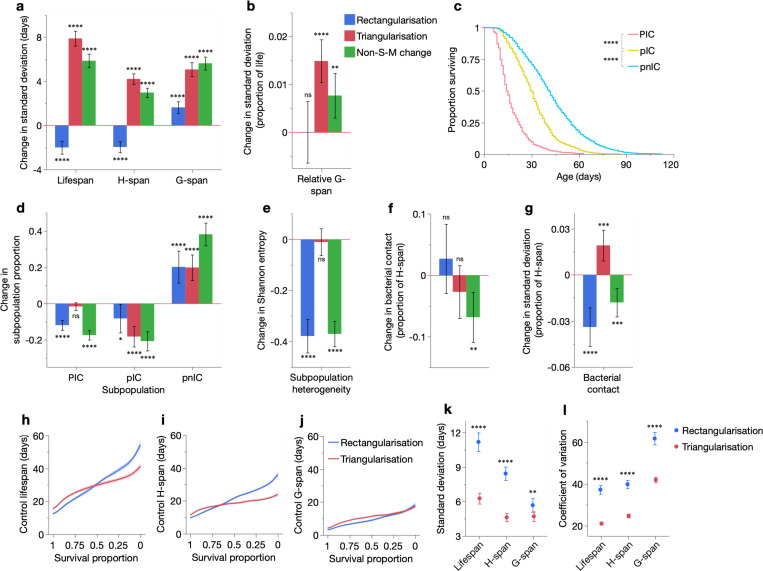
S-M^rect^ and S-M^tri^ treatments, respectively, homogenise and heterogenise the ageing process. (**a**–**b**) Mean changes in standard deviation of (**a**) lifespan, H-span, G-span and (**b**) relative G-span, for S-M^rect^, S-M^tri^ and non-S-M treatments. (**c**) Kaplan-Meier survival curves of P, pIC and pnIC individuals from all 30 cohorts (*n*=443, 991, 2144, respectively); censored individuals were excluded. Lifespans were compared statistically using the log-rank test. P: infected swollen pharynx; p: uninfected pharynx (Zhao et al., 2017). Therefore, PIC: P with intestinal colonisation, pIC: p with intestinal colonisation, pnIC: p with no intestinal colonisation (Zhang and Gems, 2026). (**d**) Mean changes in subpopulation proportion by S-M^rect^, S-M^tri^ and non-S-M treatments, and (**e**) mean changes in population heterogeneity resulting from these subpopulation proportion changes, as measured by Shannon entropy. (**f**–**g**) Mean changes in the (**f**) mean and (**g**) standard deviation of bacterial contact (proportion of H-span in contact with *E. coli* lawn) by S-M^rect^, S-M^tri^ and non-S-M treatments. (**h**–**j**) Spline smoother of mean (**h**) lifespan, (**i**) H-span and (**j**) G-span of control cohorts that underwent S-M^rect^ versus S-M^tri^ treatments, plotted over survival proportion (x-axis left: shorter-lived population members, x-axis right: longer-lived population members). 95% confidence regions are shaded. (**k**–**l**) Mean (**k**) standard deviation and (**l**) coefficient of variation of lifespan, H-span and G-span of control cohorts that underwent S-M^rect^ versus S-M^tri^ treatments (*n*=101, 162). Means were compared statistically using two-tailed Student’s t-tests; 95% confidence intervals are shown. Changes in means in **a**–**b** and **d**–**g** were compared statistically using two-tailed one sample t-tests (*H*_0_: mean change=0), showing 95% confidence intervals; Benjamini-Hochberg correction of *p* values did not change which treatments reached the 0.05 significance threshold (data not shown). In all panels: ns *p* > 0.05, * *p* ≤ 0.05, ** *p* ≤ 0.01, *** *p* ≤ 0.001, **** *p* ≤ 0.0001.

As expected, lifespan standard deviation was decreased in S-M^rect^ treatments and increased in S-M^tri^ treatments (Fig. 6a, left). This reflects the compression of mortality and extension of maximum lifespan, respectively, that occur in these demographic changes (Fig. 1c, e, f, h). The non-S-M treatments also increased lifespan standard deviation, but less than S-M^tri^ treatments, consistent with increases in both maximum and early lifespan.

Notably, inter-individual variation in H-span and G-span changed in the same directions as lifespan variation, except for an increase in G-span variation in S-M^rect^ treatments (Fig. 6a, Extended Data Fig. 5a–b). In line with these changes, relative G-span was unaltered in S-M^rect^ treatments and increased in S-M^tri^ and non-S-M treatments (Fig. 6b, Extended Data Fig. 5d–e). Normalisation of these changes to their means (coefficient of variation) revealed that respectively, S-M^rect^ and S-M^tri^ treatments disproportionately decreased and increased inter-individual variation in H-span, G-span and relative G-span (Extended Data Fig. 5c, f). These findings suggest that S-M^rect^ and S-M^tri^ treatments respectively homogenise and heterogenise the ageing process, resulting in respectively reduced and increased inequalities in the durations of health and morbidity.

These changes in H-span and G-span may reflect changes in biological ageing rate across the life course, which would stretch or compress their durations. However, individual *C. elegans* vary not only in the speed of ageing but also its trajectory. Distinct (yet isogenic) subpopulations have been characterised, which acquire organ-specific, ageing-related colonisations by the dietary *E. coli*, affecting both the pharynx and intestine (“PIC” individuals), the intestine only (“pIC” individuals), or neither organ (“pnIC” individuals) (Zhao et al., 2017, Zhao et al., 2021, Zhang and Gems, 2026) (Fig. 6c caption). These subpopulations were scored in the present dataset, by necropsies on 91% of all uncensored individuals (3,578/3,911) across the 30 cohorts. Notably, the three subpopulations had different longevities, with longer lifespans when fewer organs were bacterially colonised (Fig. 6c).

Notably, S-M^rect^ treatments primarily reduced the prevalence of the short-lived P individuals (and intermediate-lived pIC individuals), replacing them with individuals from the longest-lived pnIC subpopulation (Fig. 6d). Thus, lifespan rectangularisation here results from the redirection of individuals from shorter- to longer-lived ageing trajectories, which postpones and compresses mortality into the survival curve tail. Conversely, S-M^tri^ treatments converted only pIC individuals into pnIC individuals (Fig. 6d), thus triangularising the survival curve by maintaining early mortality while pushing maximum lifespan. Finally, the non-S-M treatments converted both P and pIC individuals to pnIC, consistent with their postponement of both early and late mortality. Therefore, these three demographic modes of lifespan extension involve distinct individual-specific changes in both the rate and trajectory of ageing.

Notably, S-M^rect^ treatments decreased two measures of subpopulation heterogeneity (Shannon entropy and Simpson’s Diversity Index) (Fig. 6e, Extended Data Fig. 5j), supporting their action as homogenising treatments. However, S-M^tri^ treatments did not change subpopulation heterogeneity, suggesting they increase inter-individual variation in the rate (H-span and G-span) but not the trajectory of ageing.

We also asked how early in life such variability changes emerge. Given ageing-related colonisation by dietary *E. coli*, and that intestinal *E. coli* load in early adulthood predicts nematode lifespan (Baeriswyl et al., 2010), we scored the proportion of H-span spent in contact with the bacterial lawn, for all individuals of the 30 cohorts in at least 3 trials. Mean bacterial contact of cohorts was largely unchanged by S-M^rect^ and S-M^tri^ treatments, and decreased by non-S-M treatments (Fig. 6f). Interestingly, however, inter-individual variation in bacterial contact was mostly decreased by S-M^rect^ treatments and increased by S-M^tri^ treatments (Fig. 6g, Extended Data Fig. 5g–i), consistent with the direction of variation change in H-span, G-span, relative G-span, disease subpopulation heterogeneity and lifespan. Taken together, these findings demonstrate that S-M correlations reflect specific changes in the inter-individual variability of the biological ageing process: homogenisation in S-M^rect^ treatments and heterogenisation in S-M^tri^ treatments. Consistent with this, the non-S-M treatments, which produce intermediate changes in lifespan variation (between S-M^rect^ and S-M^tri^ treatments), exhibited greater variation in the type of variability change seen (i.e. homogenisation or heterogenisation).

Finally, we wondered what determines whether a given life-extending intervention has an S-M^rect^ or an S-M^tri^ outcome. Intriguingly, lifespan, H-span and G-span were consistently more inter-individually variable in the control cohorts that underwent S-M^rect^ than S-M^tri^ treatments (Fig. 6h–l). This argues that demographic responses to lifespan-extending interventions depend on the existing level of variation: heterogeneous populations are more likely to undergo rectangularisation, and homogeneous populations triangularisation. Through which specific biological traits this demographic decision is made remains to be determined.

### Conservation of S-M correlation determinants in *Drosophila* and mice

Finally, we wondered whether we could explain the biological basis of S-M correlations in other species. Species differences in the ageing process and in health/morbidity definitions may argue against the conservation of nematode H-span/G-span dynamics across species. However, these dynamics could potentially be upstream of specific mechanisms and thereby follow similar biodemographic principles.

Availability of lifelong longitudinal healthspan data of other animal models is somewhat more limited than for *C. elegans*. We analysed two *Drosophila* and three mouse datasets, which despite having smaller cohort sizes and often less frequent longitudinal health measurements, importantly contained health and lifespan data from the same individuals, across multiple cohorts. We therefore similarly treated each lifespan-extending pairwise comparison between cohorts as a lifespan-extending treatment, split them into S-M^rect^ and S-M^tri^ treatments, and compared their effects on lifespan, H-span, G-span and relative G-span.

**Fig. 7.**
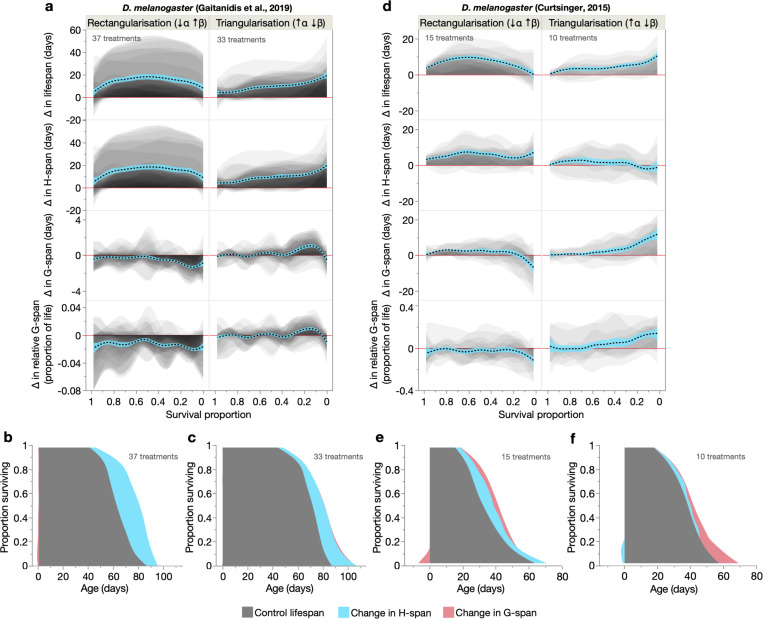
Health and morbidity profiles of S-M correlations in *Drosophila melanogaster*. (**a**, **d**) Shaded and overlaid (JMP transparency=0.05) area plots for lifespan-extending S-M^rect^ and S-M^tri^ treatments, of changes in lifespan, H-span, G-span and relative G-span. These shaded changes are plotted over survival proportion (x-axis left: shorter-lived population members, x-axis right: longer-lived population members). Red line: y=0, below which the lifespan-extending treatment shortens that trait. Dashed black line: summary spline smoother (lambda=0.05) of the treatment changes, showing its 95% confidence region in blue. The number of treatments for S-M type is annotated. (**b**, **c**, **e**, **f**) Empirical summary figures of how S-M^rect^ and S-M^tri^ treatments extend lifespan through additive changes in H-span and G-span (spline smoother of each, stacked on spline smoother of control lifespan), displayed in a shaded survival curve-like format. Shortening of H-span and G-span is represented as a negative change relative to Age=0.

The *D. melanogaster* datasets measured age-related locomotory decline in 14 cohorts undergoing different dietary regimes (protein:carbohydrate ratio, and curcumin and fruit extract supplementation; both sexes) (Gaitanidis et al., 2019), and reproductive ageing (age-decline in fecundity) in females of 9 laboratory-cultured and wild-caught lines (Curtsinger, 2015) (Supplementary Tables 6, 7). In both datasets, S-M^rect^ treatments primarily increased H-span, in short- to intermediate-lived population members (Fig. 7a–b, d–e). G-span was decreased in the longest-lived individuals, but only modestly. Thus, as in our nematode cohorts, it is primarily H-span expansion that rectangularises the survival curve. Also similar to the nematode data, the S-M^tri^ treatments generally increased H-span and G-span in longer-lived population members (except H-span in the reproductive ageing dataset) (Fig. 7a, c, d, f). Furthermore, in both datasets, relative G-span increased marginally in S-M^tri^ treatments; however, the S-M^rect^ treatments trended towards morbidity compression (Fig. 7a, d, bottom). The biological determinants of these S-M correlations in *Drosophila* therefore overlap with those in *C. elegans*.

**Fig. 8.**
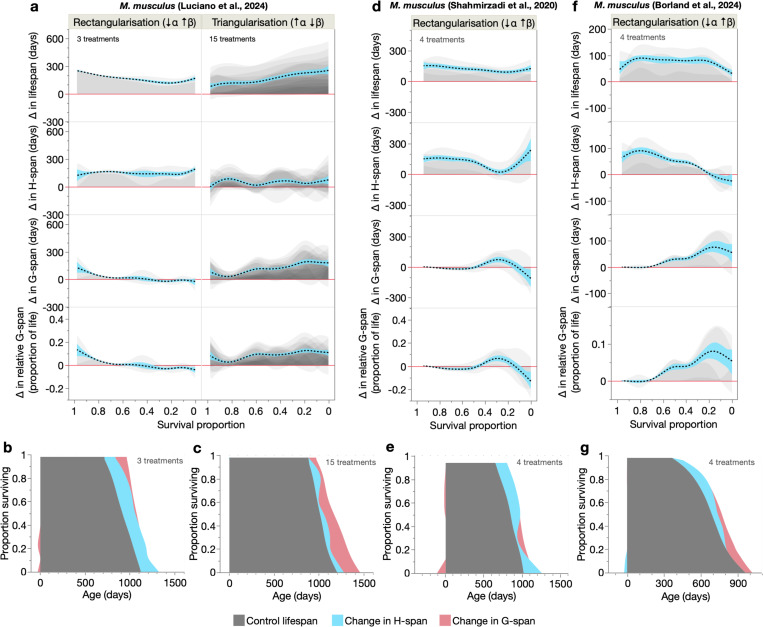
Health and morbidity profiles of S-M correlations in mice. (**a**, **d**, **f**) Shaded and overlaid (JMP transparency=0.05) area plots for lifespan-extending S-M^rect^ and S-M^tri^ treatments, of changes in lifespan, H-span, G-span and relative G-span. These shaded changes are plotted over survival proportion (x-axis left: shorter-lived population members, x-axis right: longer-lived population members). Red line: y=0, below which the lifespan-extending treatment shortens that trait. Dashed black line: summary spline smoother (lambda=0.05) of the treatment changes, showing its 95% confidence region in blue. The number of treatments for S-M type is annotated. (**b**, **c**, **e**, **g**) Summary figures of how S-M^rect^ and S-M^tri^ treatments extend lifespan through additive changes in H-span and G-span, displayed in a shaded survival curve-like format. Shortening of H-span and G-span is represented as a negative change relative to Age=0.

We next examined the three mouse cohorts, which measured longitudinal age changes in a 32-item fragility index in response to caloric restriction and intermittent fasting (Luciano et al., 2024) (7 cohorts), and 31-item frailty indexes given alpha-ketoglutarate treatment (Shahmirzadi et al., 2020) (4 cohorts) and mutation of RNA polymerase III (Borland et al., 2024) (4 cohorts) (Supplementary Tables 8–10). The first dataset yielded primarily S-M^tri^ treatments (Fig. 8a), consistent with reports of β reduction by dietary restriction in rodents (Nakagawa et al., 2012, Simons et al., 2013), while the latter two datasets yielded S-M^rect^ treatments (Fig. 8d, f). In all three datasets, S-M^rect^ treatments primarily increased H-span, which occurred mainly in short-lived population members (Fig. 8a–b, d–g), thus rectangularising the survival curve as in the nematode and *Drosophila* datasets. However, in the dietary restriction dataset, H-span also increased in longer-lived population members and G-span increased mildly in the shortest-lived, suggesting that here rectangularisation arises from combined changes in H-span and G-span (Fig. 8a–b). Survival curve triangularisation was driven alone by G-span increases in longer-lived population members (Fig. 8a, c). Across these datasets, relative G-span was either unchanged or increased, consistent with the greater frequency of morbidity expansion in our nematode treatments.

Taken together, these nematode, fruit fly and mouse cohorts exhibit both common and distinct biological bases of the S-M correlation. Despite species differences in the ageing process and H-span/G-span definitions, rectangularisation of survival curves was driven by H-span expansion in shorter-lived population members, and triangularisation by G-span expansion in longer-lived population members. These similarities may reflect conserved demographic dynamics of how durations of health and morbidity vary between individuals, and respond to lifespan-extending interventions. Indeed, strikingly, as in our nematode dataset (Fig. 6h–l), lifespan variation was consistently higher in the control cohorts that underwent S-M^rect^ than S-M^tri^ treatments, in the two *Drosophila* and one mouse datasets that had both forms of S-M correlation (Extended Data Fig. 6). This suggests another species-independent principle of demographic lifespan extension, wherein heterogeneous populations undergo rectangularisation and homogenous populations triangularisation.

## Discussion

**Fig. 9.**
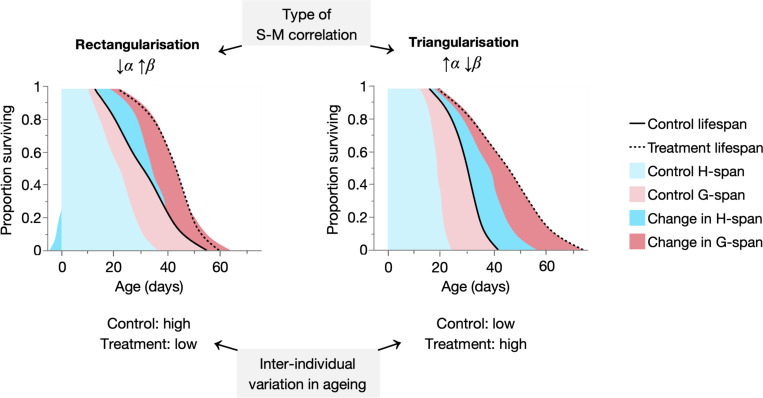
Summary schematic: rectangularisation and triangularisation decrease and increase variation in ageing, respectively. Empirical summary figures depicting how the two types of S-M correlation (rectangularisation and triangularisation) extend lifespan through additive changes in H-span and G-span, in a shaded survival curve-like format. Rectangularisation results from increases in H-span in shorter-lived population members, accompanied by relatively equal G-span increases across the population, whereas triangularisation results from increases in both H-span and G-span in longer-lived population members. These changes (and others; see Fig. 6a, Extended Data Fig. 5) reflect decreases and increases, respectively, in the inter-individual (within-population) variability of the ageing process by interventions that rectangularise and triangularise the survival curve. Notably, such interventions cause rectangularisation when existing variation (in control cohorts) is high (thereby reducing it), and triangularisation when the existing variation is low (thereby increasing it).

Here, we used *C. elegans* to experimentally determine the biological basis of the Strehler-Mildvan correlation between parameters of the Gompertz mortality model. By studying pairwise instances of the S-M correlation (between two cohorts), corresponding to life-extending rectangularising (S-M^rect^) and triangularising (S-M^tri^) survival curve transformations, we resolved tractable, causal relationships between biological ageing and S-M mortality patterns. S-M^rect^ correlations arose from healthspan changes in shorter-lived population members, whereas S-M^tri^ correlations arose from healthspan and gerospan changes in longer-lived population members (Fig. 9). Importantly, S-M correlations either homogenised (if S-M^rect^) or heterogenised (if S-M^tri^) the biological ageing process between population members, depending on the degree of existing heterogeneity (Fig. 9).

These findings provide an empirical explanation for the S-M correlation, and data for validation and development of theoretical ageing models that predict the correlation (Strehler and Mildvan, 1960, Gavrilov and Gavrilova, 1991, Li and Anderson, 2015), including the original S-M general theory of mortality and ageing (Strehler and Mildvan, 1960). The data also explain the biological processes underlying *absences* of the S-M correlation, which have challenged theoretical models that expect this correlation.

Our results favour inter-individual heterogeneity explanations of the S-M correlation over those assuming intra-individual trade-offs between health and ageing (Vaupel and Yashin, 1985, Yashin et al., 2001, Hawkes et al., 2012). Trade-off explanations arise from theoretical interpretations of model parameters, such as the conventional equation of α and β with ageing-independent processes and biological ageing rate, respectively (Sacher, 1977, Finch et al., 1990). However, our work argues against these interpretations (in fact, inverting them (Zhang and Gems, 2026)); indeed, that healthspan extensions cause lifespan rectangularisation is a further argument against traditional interpretations of increased β as accelerated ageing. We also show that within isogenic populations, biological ageing rates and trajectories vary greatly. Mortality patterns may therefore be more appropriately studied as inter-individual *distributions* of biological ageing processes. Understanding such variability distributions, which appear to be stochastic in origin, yet reproducible (Finch and Kirkwood, 2000), are likely to be foundational for understanding and intervening in ageing.

Why is the S-M correlation so common across conditions and species? Our findings suggest that inter-individual variation, present in all populations, controls fundamental biodemographic dynamics of mortality. In all species examined (nematodes, flies and mice), the amount of cohort heterogeneity predicted whether lifespan-extending interventions would rectangularise or triangularise survival. This may reflect the greater likelihood of homogeneous populations to lose (rather than further increase) homogeneity, and vice versa for heterogeneous populations, somewhat akin to the principle of regression towards the mean (Galton, 1886). Additionally, both rectangularisation and triangularisation involve changes that affect individual longevity to different extents, consistent with known individual-specificities of many lifespan- and health-modulating interventions (Di Francesco et al., 2024, Kuchel et al., 2025).

We observed both similarities and differences in the healthspan and gerospan dynamics underlying the S-M correlation between nematodes, flies and mice. In common was healthspan expansion in shorter-lived population members in rectangularisation, and gerospan expansion in longer-lived population members in triangularisation, although these patterns varied in magnitude. Such cross-species comparisons are limited by data availability and resolution (e.g. population size and health measurement frequency), and complicated by differing health/morbidity definitions, though larger longitudinal studies capturing multiple ageing phenotypes are underway (Palliyaguru et al., 2021, Creevy et al., 2022).

Are our findings relevant to human populations? Human lifespan has rectangularised over the last two centuries, given reductions in early-life mortality by communicable diseases (Riley, 2001), concentrating deaths into later ages (Fries, 1980, Riffe et al., 2020). This may extend lifespan mainly through healthspan increases in shorter-lived population members, by preventing premature death in these otherwise healthy individuals. Notably we observed this, as shorter-lived, bacterially-infected subpopulations were converted into longer-lived, uninfected subpopulations, in our S-M^rect^ nematode treatments.

Importantly, this homogenisation of human populations (towards ageing-related deaths) has slowed further reductions in early mortality (Christensen et al., 2009), while maximum lifespan has gradually increased in more recent decades, reflecting medical advances against ageing-related diseases (Bongaarts, 2005, Christensen et al., 2009, Bergeron-Boucher et al., 2015). In fact, decelerations of mortality rate increase at advanced ages (Vaupel et al., 1998, Barbi et al., 2018) (although this remains debated (Newman, 2018, Dang et al., 2023)) and increased inter-individual variation in human health (Seaman et al., 2020) have been observed. Intriguingly, these changes are reminiscent of lifespan triangularisation, suggesting that human survival may be experiencing a demographic transition from rectangularisation to triangularisation. Supporting this possibility, we observed that lifespan extension of homogeneous (rectangularised) populations occurs through triangularisation. Our S-M^tri^ nematode treatments increased both healthspan and gerospan in the survival curve tail, and produced the smallest increases in relative morbidity, compared to the other modes of lifespan extension. Whether similar health and morbidity dynamics could underly human triangularisation remains to be seen.

## Methods

### *C. elegans* culture and strains

*C. elegans* were maintained at 20°C using standard protocols (Brenner, 1974), on Nematode Growth Medium (NGM) plates seeded 2 days before use with a bacterial food source (*Escherichia coli* OP50). Floxuridine (5-fluoro-2-deoxyuridine), sometimes used to block progeny production, was not used in this study. Nematode strains used were: N2 (wild-type, hermaphrodite stock (Zhao et al., 2019)), GA1959 *daf-2(m577) III*, GA1960 *daf-2(e1368) III*, GA1928 *daf-2(e1370) III*, and GA1952 *daf-16(mgDf50) I*. All strains were raised from egg at 20°C on live *E. coli*, and transferred at L4 stage to the appropriate experimental conditions (15°C, 20°C or 25°C; with or without carbenicillin). Carbenicillin solution was added topically to plates one day before adding animals (further details below).

### Ageing cohorts and lifespan scoring

Nematodes were cultured throughout life in 60 mm Petri dishes (containing 10 mL of NGM) seeded with approximately 80 μL of *E. coli*, and where relevant, treated with 80 μL of 500 mM carbenicillin (Fisher Scientific Ltd, catalogue no. 12737149). In each trial, at L4 stage (time 0 in all analyses), 25 animals were placed on a plate, with two plates per condition. Animals were transferred every two days during the reproductive period. Following the end of egg laying, animals were transferred to individual wells of 24-well tissue culture plates, containing 2 mL of NGM and seeded with 3.5 μL of *E. coli* OP50, and where relevant, treated with 16 μL of 500 mM carbenicillin. Animals were subsequently transferred to fresh 24-well plates monthly, before media desiccation (plates were sealed with parafilm to delay desiccation, and to prevent bacterial/fungal contamination). Scoring of survival was performed every 2–3 days alongside scoring of locomotory class, and necropsy at death (described below). Animals showing no movement were gently touched with a platinum wire (worm pick) on the head and/or tail; those that showed no movement at all in response were scored as dead. Animals that died due to desiccation on the Petri dish wall, internal hatching of larvae, or rupture of internal tissues through the vulva, or that became contaminated by non-*E. coli* bacteria or fungi, or could not be found, were censored.

### Quantification of locomotory decline with age

Locomotory health class (belonging to H-span or G-span) was scored by classifying individuals into one of three classes, adapted from earlier systems (Hosono et al., 1980, Herndon et al., 2002): A – sinusoidal locomotion; B – non-sinusoidal locomotion; C – no locomotion. To accurately determine locomotory class, animals were gently touched on the tail with a platinum wire worm pick for up to 20 seconds to induce an escape response that reveals movement capacity (rather than behavioural preference (Hahm et al., 2015)), and additionally on the head as a final check. The duration spent in A class was defined as H-span, and the summed duration spent in B and C classes as G-span. Here, B and C classes were summed to improve data tractability, and to provide a definition of G-span that captures both early and late-stage functional declines.

### Necropsy analysis

Necropsy to define patterns of *E. coli*-associated pathology was performed by examining fresh corpses under a Leica MZ8 stereomicroscope (50x magnification). Scoring of swollen, bacterially-infected pharynxes (P), and uninfected, atrophied pharynxes (p) was performed as previously described (Zhao et al., 2017). Intestinal colonisation (IC) with *E. coli* was scored where severe bacterial accumulation was observed in the anterior and/or posterior intestine. Such colonisation presented as extreme lumenal distension by proliferating bacteria and/or colonisation of the intestine beyond the lumenal barrier, with concomitant intestinal tissue degeneration. In pharyngeal and intestinal tissues, sites of bacterial colonisation exhibit a yellow-brown colour (like that of the *E. coli* lawn and colocalising with RFP-labelled *E. coli*), translucent and uniform texture (loss of healthy tissue structures that otherwise appear dark, granular and opaque), and swollen/distended morphology (extensive proliferation of live *E. coli*) (Zhang and Gems, 2026).

### Bacterial contact scoring

Bacterial contact was scored every 1–2 days. Animals were scored as in contact with bacteria if they were observed inside the lawn at first sighting; each animal was checked once during each scoring session. Importantly, plates were handled carefully to avoid sudden movements or knocks that could startle animals and affect their location within their wells. Notably, animals were often observed to be stationary at the edge of the lawn, resting with their heads outside of the lawn; these animals were scored as not in contact with the bacteria. Accordingly, animals resting with their heads inside the lawn but tail outside were scored as in contact with the bacteria. Where nematode tracks led to growth of *E. coli* outside of the central lawn, nematode interaction with these colonies were treated the same as for the central lawn. If the media surface approached complete coverage by *E. coli*, animals were transferred to fresh wells to allow detection of bacterial avoidance.

For analysis, bacterial contact was quantified as the proportion of locomotory healthspan (A-span) spent in contact with the bacteria, to avoid confounding effects of locomotory decline on the locomotion-dependent bacterial contact phenotype. Because scoring commenced in most cases after the reproductive period (when animals were moved to 24-well plates), A-span was here modified by subtracting the number of days during which bacterial contact was not scored.

### Statistics, software and data handling

Lifespan, locomotory and necropsy data for N2, *daf-2(m577)*, *daf-2(e1368)* and *daf-2(e1370)* cohorts have been analysed elsewhere (Zhang and Gems, 2026), but not data for *daf-16(mgDf50)* cohorts and bacterial contact of all cohorts. Statistical tests and figure construction were performed in JMP Pro (SAS Institute, Inc.), except for Gompertz parameter estimation and assessment of statistical differences between them, which were performed using WinModest (Pletcher, 1999). Right censors were included in all Kaplan-Meier and WinModest analyses, and excluded from others. Specific statistical tests and associated methodological details are described in the respective figure/table captions. Notation of statistical significance in all figures is as follows: * *p* ≤ 0.05, ** *p* ≤ 0.01, *** *p* ≤ 0.001, **** *p* ≤ 0.0001. The measure of subpopulation heterogeneity, Shannon entropy, was calculated as –([P proportion ⋅ log(P proportion)] + [pIC proportion ⋅ log(pIC proportion)] + [pnIC proportion ⋅ log(pnIC proportion)]), and an alternative measure, Simpson’s Diversity Index, was calculated as 1 – [(P proportion)^2^ + (pIC proportion)^2^ + (pnIC proportion)^2^].

### *Drosophila* data

Drosophila data were obtained from Gaitanidis et al. (2019) and Curtsinger (2015). H-span and G-span in our reanalysis corresponds to “health-span” and “ill-span” in Gaitanidis et al. The Curtsinger data were extracted from Figure S3 using Automeris.io, producing 100 ‘pseudoindividuals’ that are representative of their survival proportion value (true cohort sizes can be found in the original study), where H-span and G-span respectively correspond to the number of days of life with at least one, or zero eggs laid. Days before day 8 were excluded due to low fecundity arising from reproductive development rather than ageing. In all datasets, the direction of changes in lifespan, H-span and G-span was always calculated in the lifespan-increasing direction (i.e. change = longer-lived cohort – shorter-lived cohort).

### Mouse data

Mouse data were obtained from Luciano et al. (2024), Shahmirzadi et al. (2020), and Borland et al. (2024). In these datasets, G-span onset was defined as the first age at which frailty or frailty index reached or exceeded a threshold value: 0.25 (fragility index) for Luciano et al. and 0.2 (frailty index) for Shahmirzadi et al. and Borland et al., when a spline smoother (lambda=0.05) was fit through each individual’s fragility/frailty index values, starting from a theoretical 0 value at 100 days (approximate age of maturity, prior to ageing). The thresholds were selected to reflect a notable number of simultaneous ageing-related deficits (i.e. not too low) and to maximise the number of individuals that reached it within their lifetimes (i.e. not too high). In the data from Luciano et al., individuals that went missing, failed to recover from anaesthesia, or were discarded were included in survival and Gompertz analysis as right censors. In all datasets, the direction of changes in lifespan, H-span and G-span was always calculated in the lifespan-increasing direction (i.e. change = longer-lived cohort – shorter-lived cohort).

## Supplementary Material

Supplement 1

Supplement 2

Supplement 3

Supplement 4

## Data Availability

Raw data for all nematode, *Drosophila* and mouse cohorts are provided.
