# Supplementary material for: The Strehler-Mildvan mortality correlation arises from changes in the variability of ageing
Source: bioRxiv. 2026 Feb 17:2026.02.15.705972. Preprint. [Version 1] doi: 10.64898/2026.02.15.705972 (PMC12934673; doi:10.64898/2026.02.15.705972)
Supplement: Supplement 1 [file media-1.docx]

Supplementary Information

**The Strehler-Mildvan mortality correlation arises from changes in the variability of ageing**

Contents Summary

**Extended Data Figure 1.** The Gompertz model provides an appropriate fit for the 30 cohorts.

**Extended Data Figure 2.** Survival curves of the pooled and individual trial data for the 30 cohorts.

**Extended Data Figure 3.** The treatment-wide S-M correlation is not an artifact of fitting Gompertz to small lifespan changes.

**Extended Data Figure 4.** Different demographic modes of morbidity compression.

**Extended Data Figure 5.** Effects of S-M and non-S-M treatments on variation in the ageing process.

**Extended Data Figure 6.** Greater lifespan variation in cohorts undergoing survival curve rectangularisation than triangularisation.


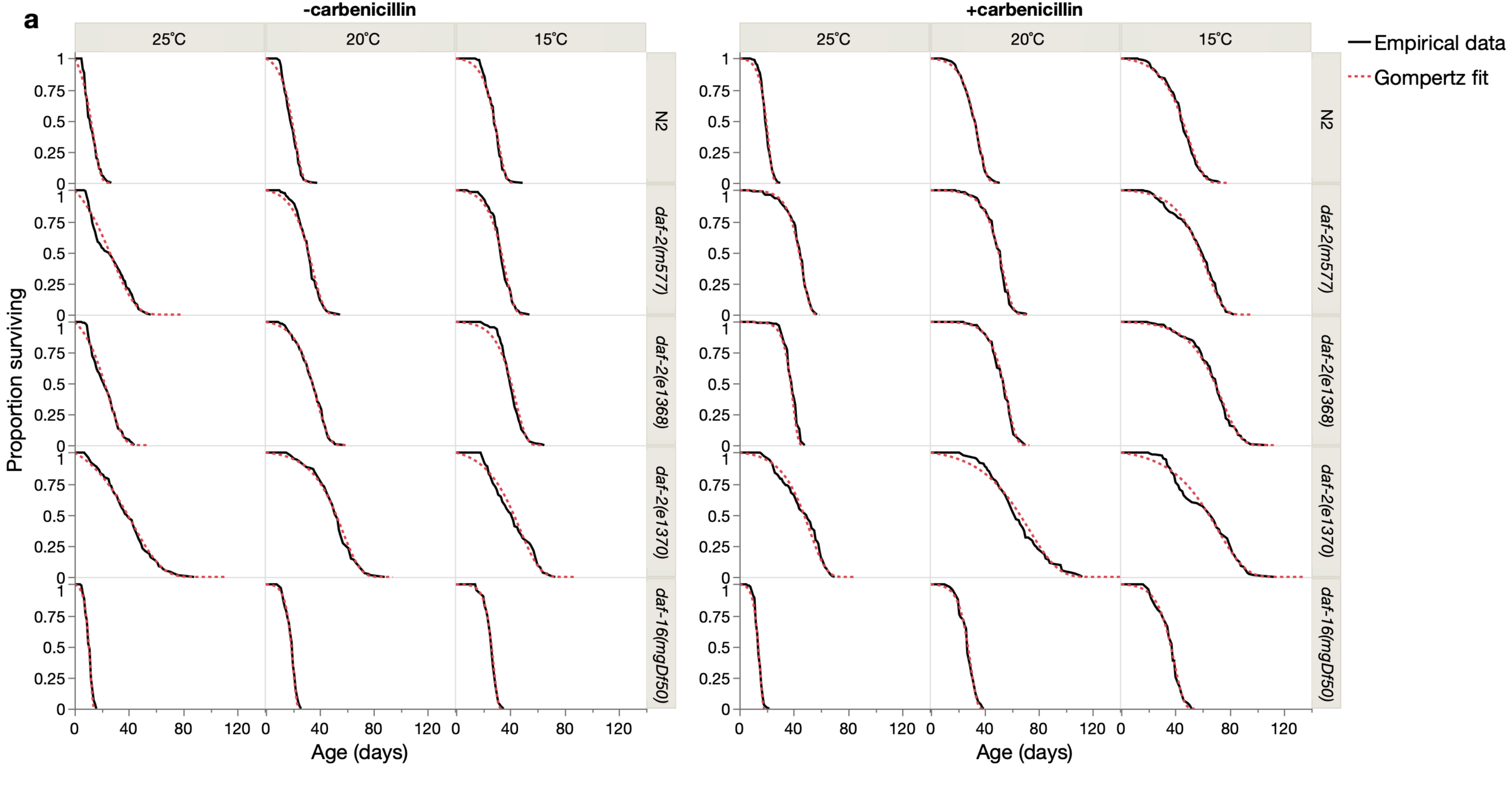

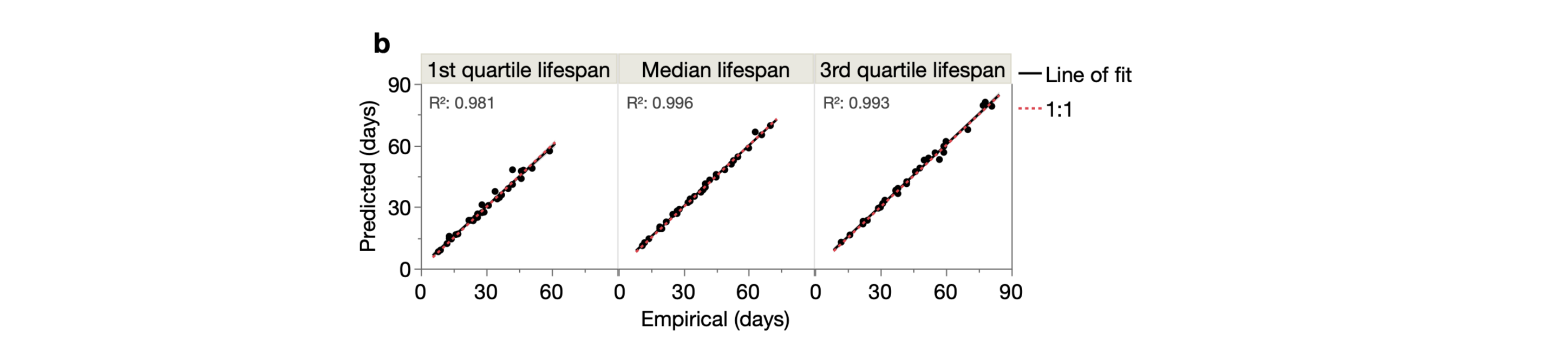


**Extended Data Figure 1.| The Gompertz model provides an appropriate fit for the 30 cohorts.** (**a**) Overlay of empirical and idealised Gompertz curves for each of the 30 cohorts (left panel: cohorts without carbenicillin; right panel: carbenicillin-treated cohorts). (**b**) Linear regressions between true (empirical) and predicted (from the Gompertz fit) lifespan measures, across the 30 cohorts. First quartile, median and third quartile lifespans are the ages at which survival proportion equals, respectively, 0.75, 0.5 and 0.25; the results indicate strong prediction of overall survival curve shape (i.e. covering ages of early, middle and late deaths) by the Gompertz model for these cohorts. 95% confidence regions around the regression fits are shaded (barely visible).


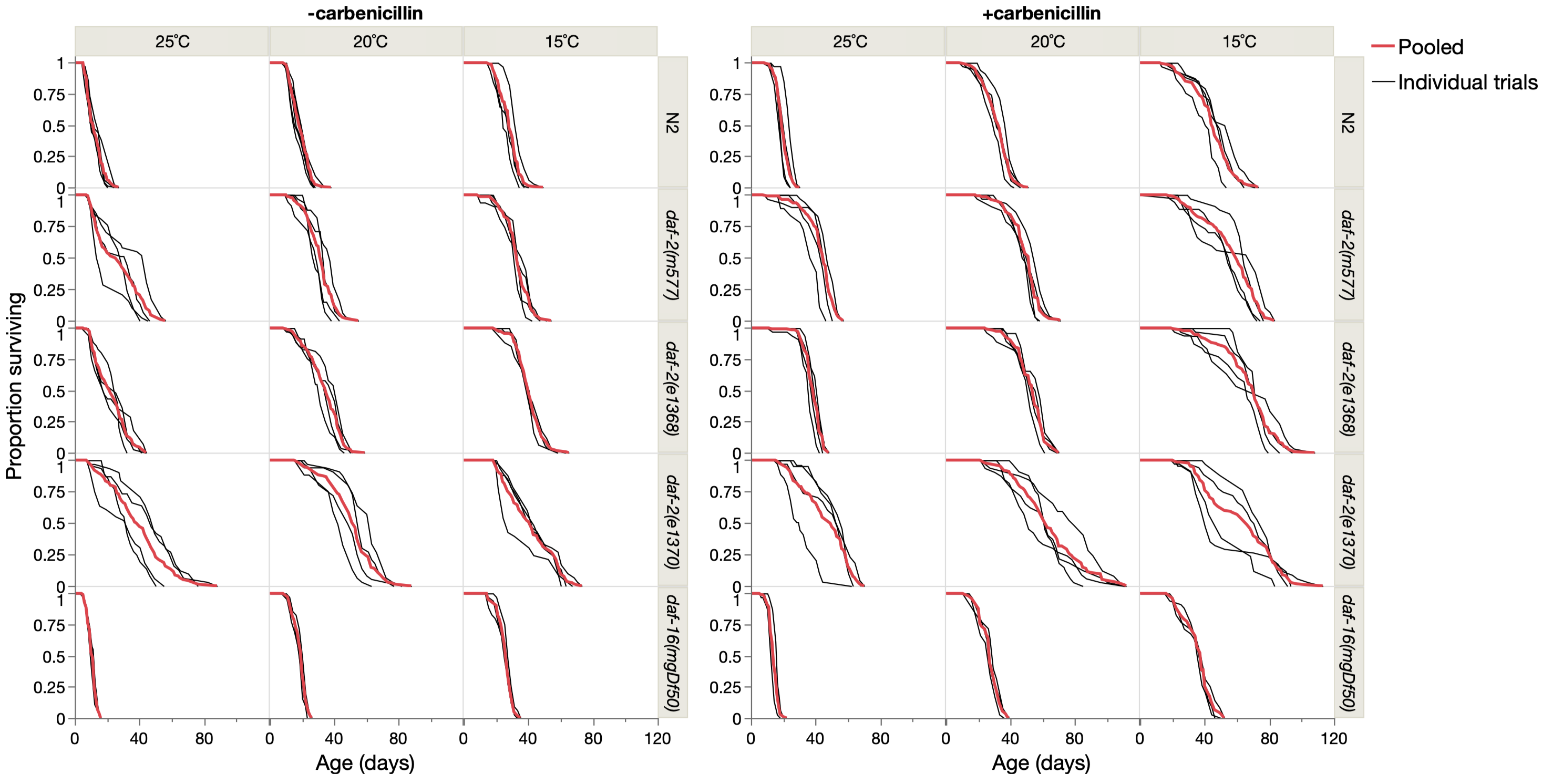


**Extended Data Figure 2. Survival curves of the pooled and individual trial data for the 30 cohorts.** Left panel: cohorts without carbenicillin; right panel: carbenicillin-treated cohorts. Survival proportions are from Kaplan-Meier survival analysis in JMP, with right-censoring. All cohorts have at least 3 individual trials. Statistical details of the individual and pooled trials are provided in Supplementary Table 1.


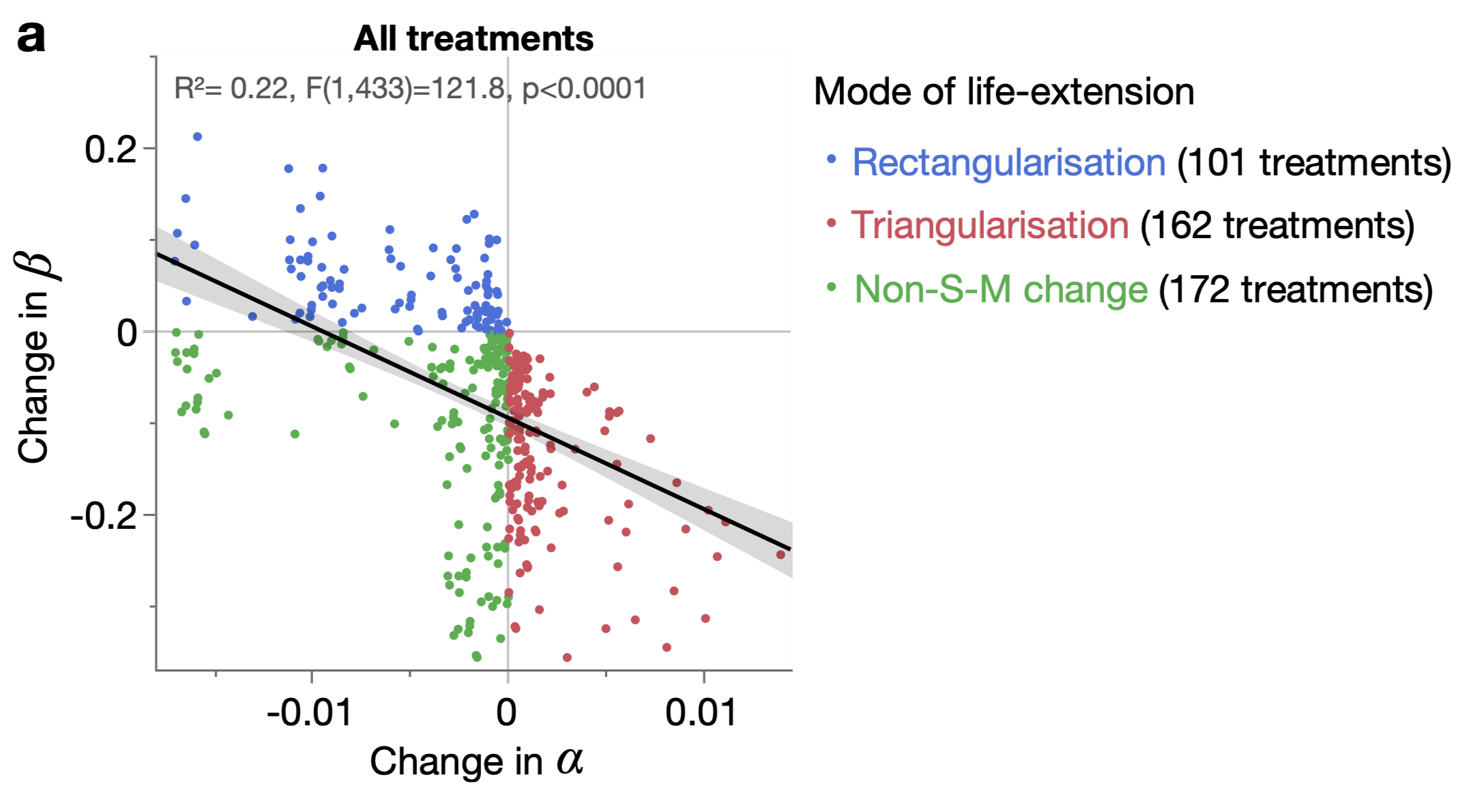

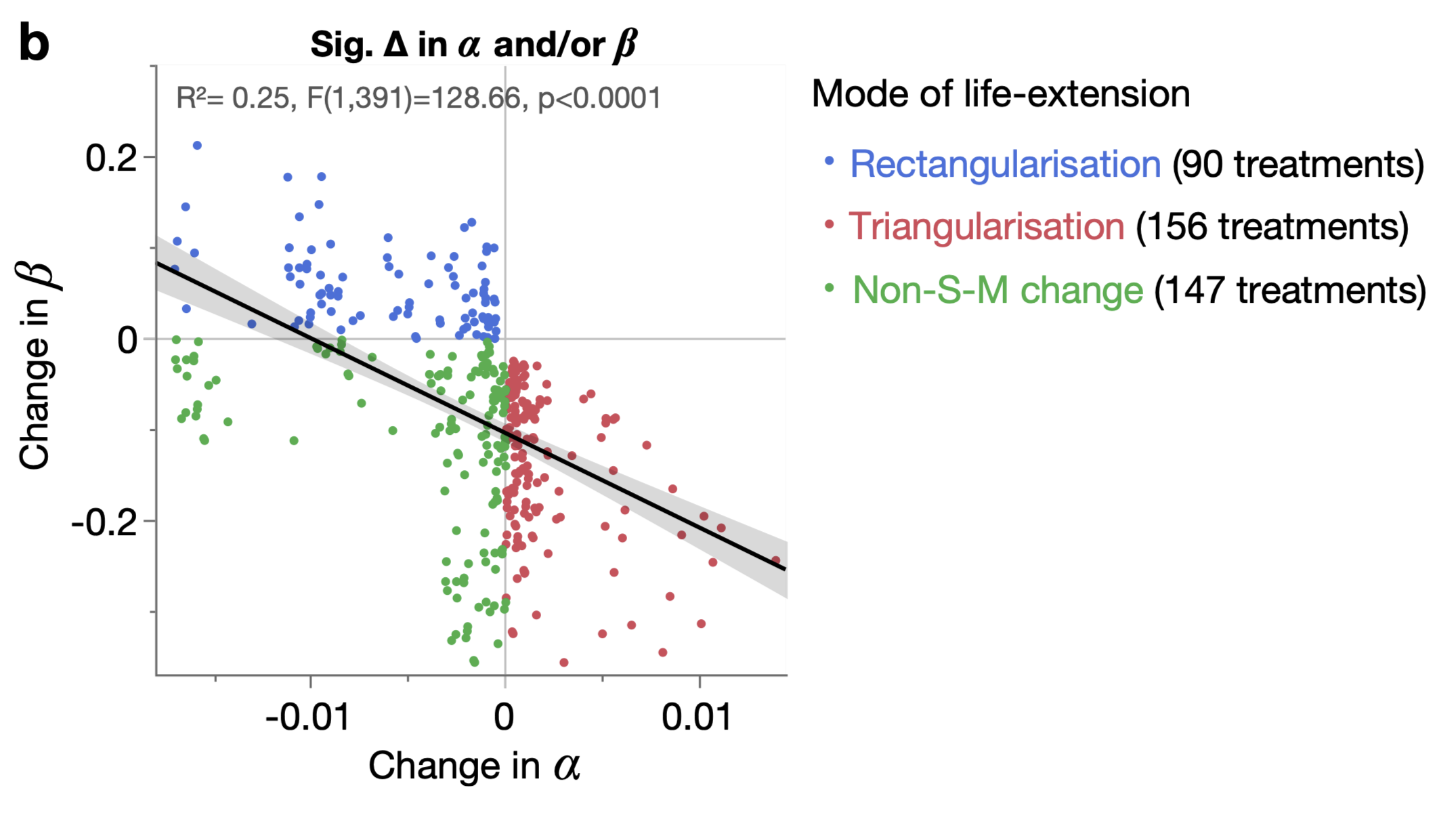

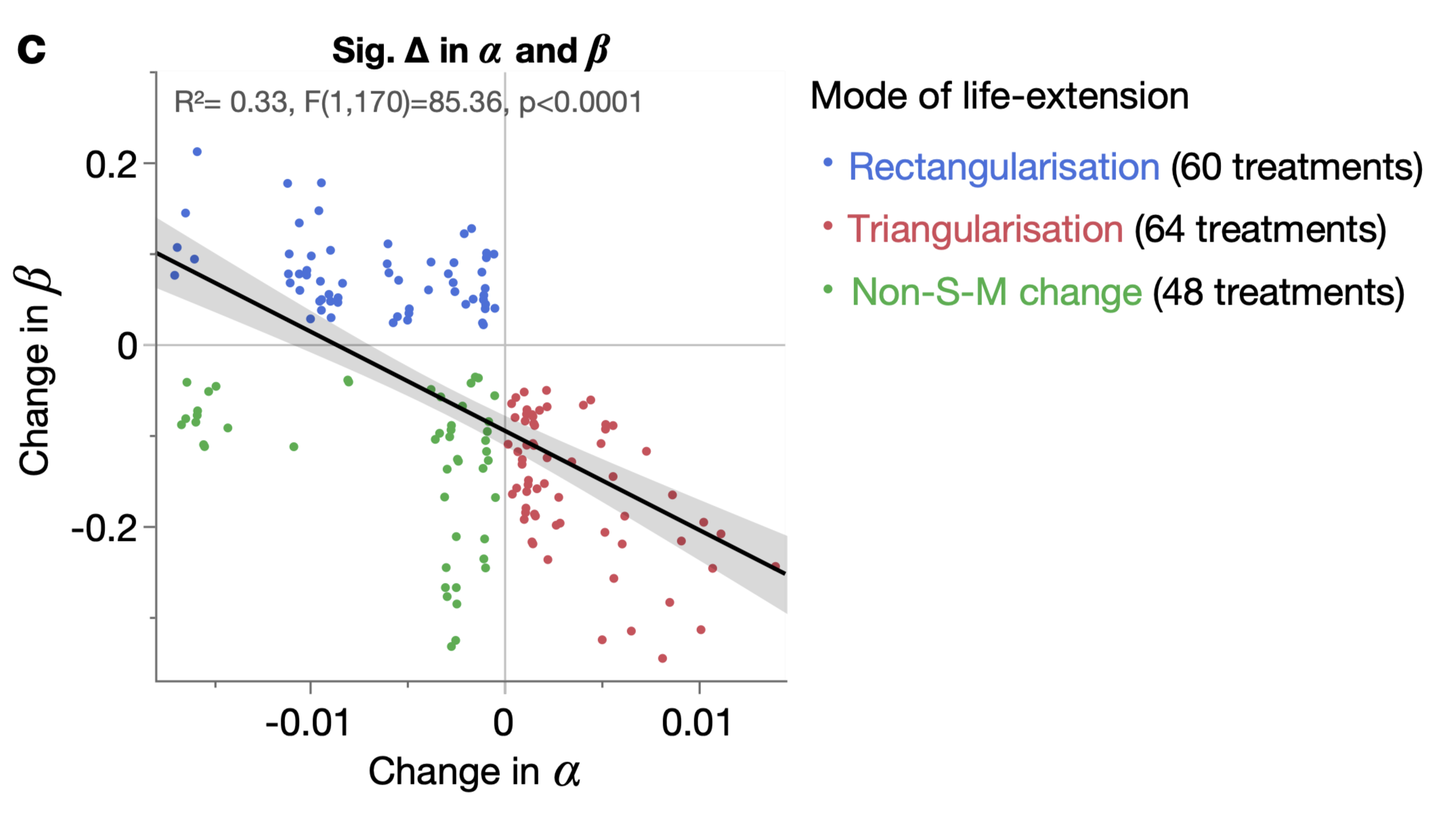

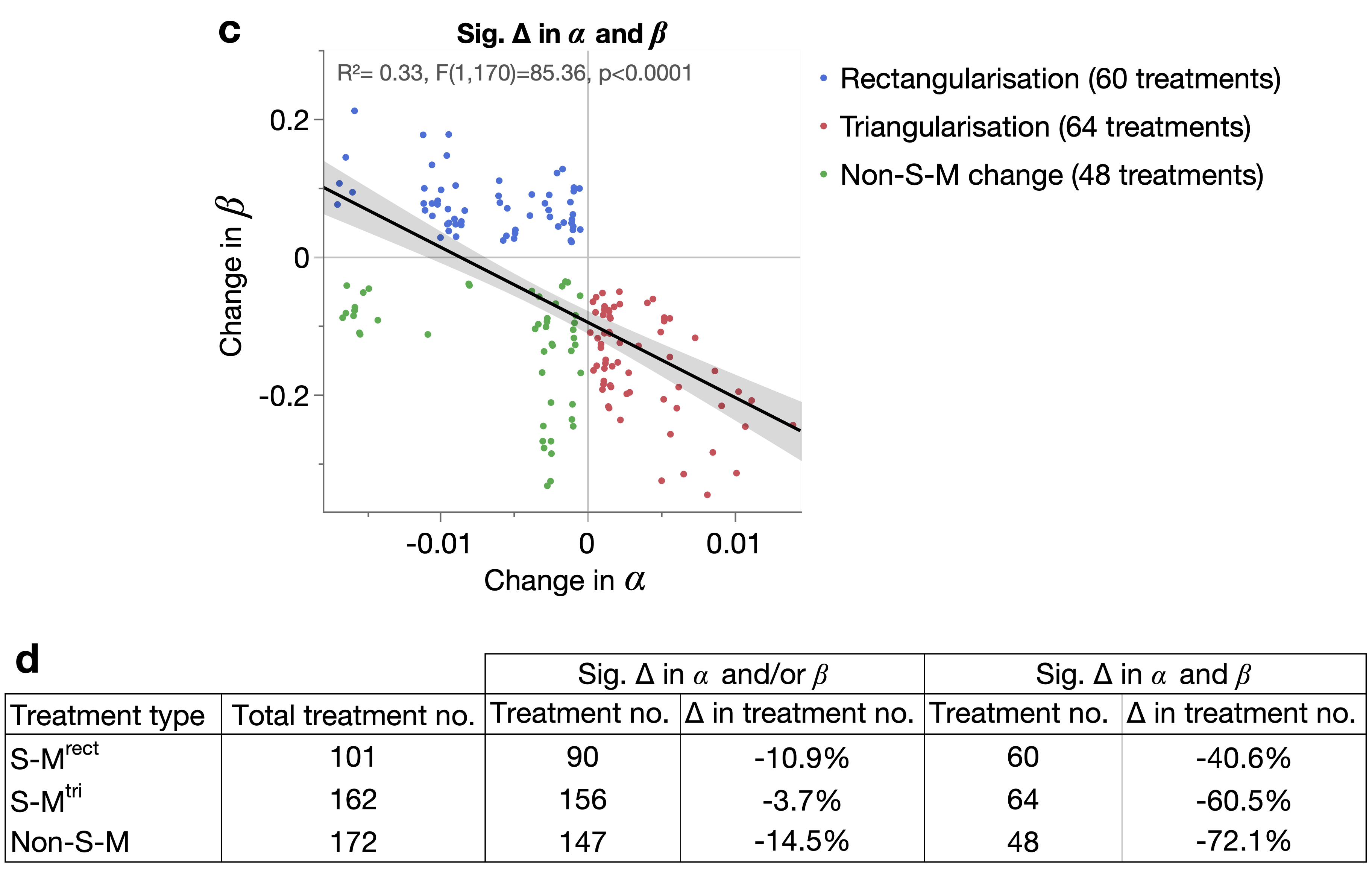


**Extended Data Figure 3. The treatment-wide S-M correlation is not an artifact of fitting Gompertz to small lifespan changes.** Least-squares linear regressions of changes in *β* over the corresponding changes in *α*, for (**a**) all 435 pairwise treatments, (**b**) only treatments in which at least one of *α* and *β* are statistically significantly changed (likelihood ratio test, Benjamini-Hochberg corrected *p*<0.05), and (**c**) only treatments in which both *α* and *β* are statistically significantly changed. Change direction was always determined based on the longer-lived cohort minus shorter-lived cohort, such that all changes reflect the effects of life-extension. The relationships were assessed by F-tests; 95% confidence regions shaded. (**d**) Summary table of the number of treatments and percent change in this number for the conditions in **b** and **c**, relative to the total number of treatments.


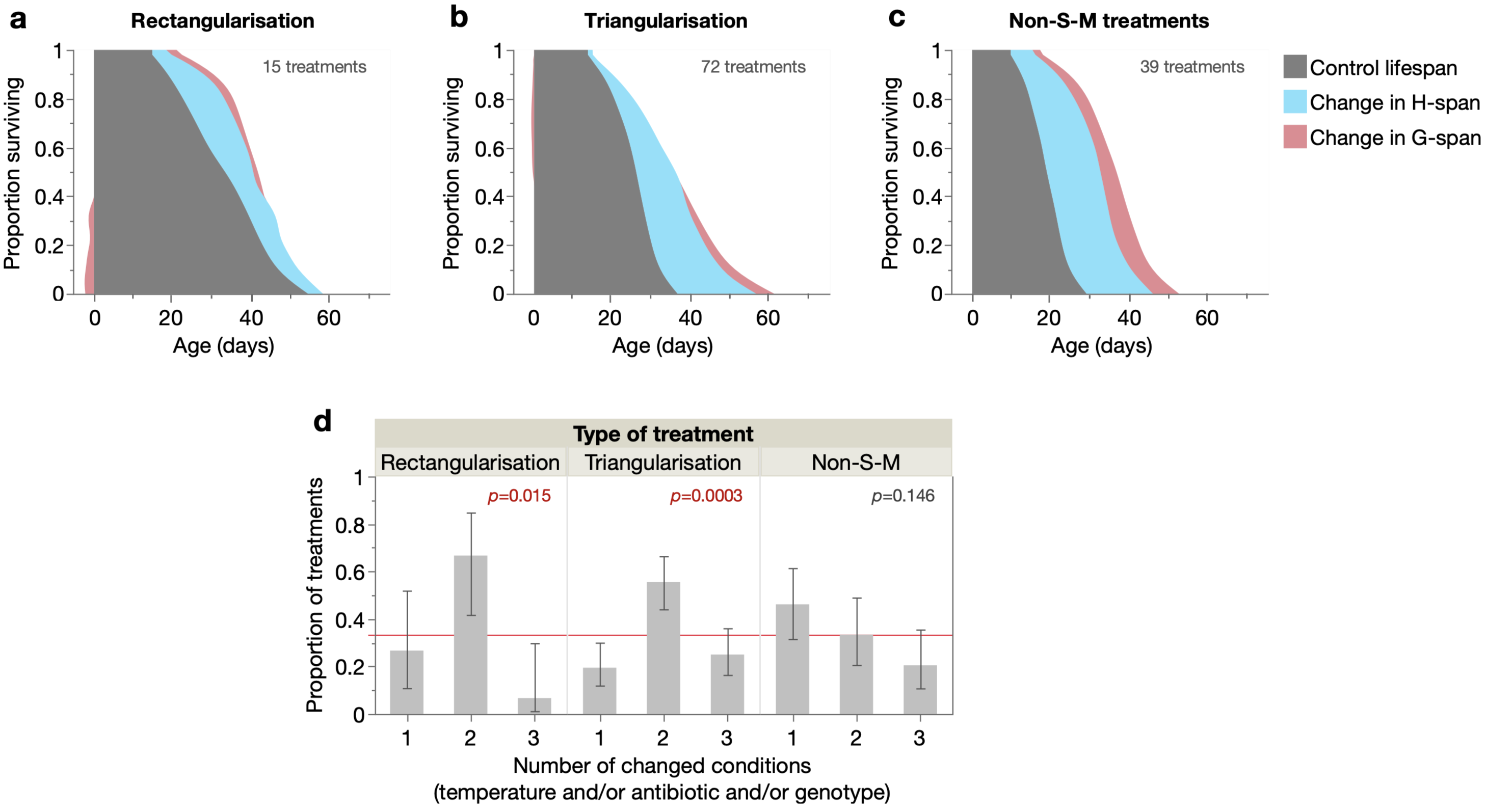


**Extended Data Figure 4. Different demographic modes of morbidity compression.** (**a**–**c**) Empirical summary figures of how S-M^rect^, S-M^tri^ and non-S-M treatments that compress morbidity (decrease relative G-span) extend lifespan through additive changes in H-span and G-span (spline smoother of each, stacked on spline smoother of control lifespan), displayed in a shaded survival curve-like format. Mild shortening of G-span in the longest-lived population members in S-M^rect^ treatments and shorter-lived population members in S-M^tri^ treatments is represented as negative changes relative to Age=0. (**d**) Proportions of the relative G-span-reducing S-M^rect^, S-M^tri^ and non-S-M treatments that arise from one, two or three changes in experiment condition (temperature, ± carbenicillin, genotype). Proportions within each panel sum to 1, and proportions above or below the red line (expected proportion under null hypothesis: 0.33) indicate enrichment or depletion of that number of changed conditions. 95% confidence intervals are shown and Pearson chi-square goodness-of-fit tests were run for each panel (*p* values annotated), with sample sizes of, S-M^rect^: 15 treatments, S-M^tri^: 72 treatments, and non-S-M: 39 treatments.

**
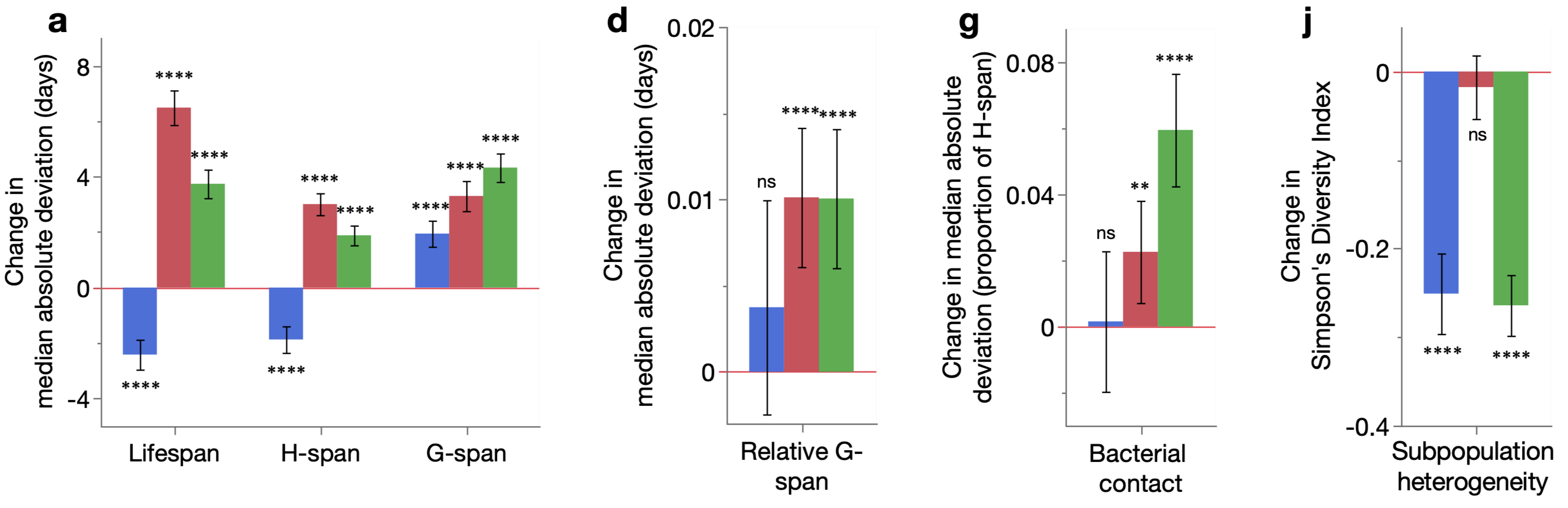
** **
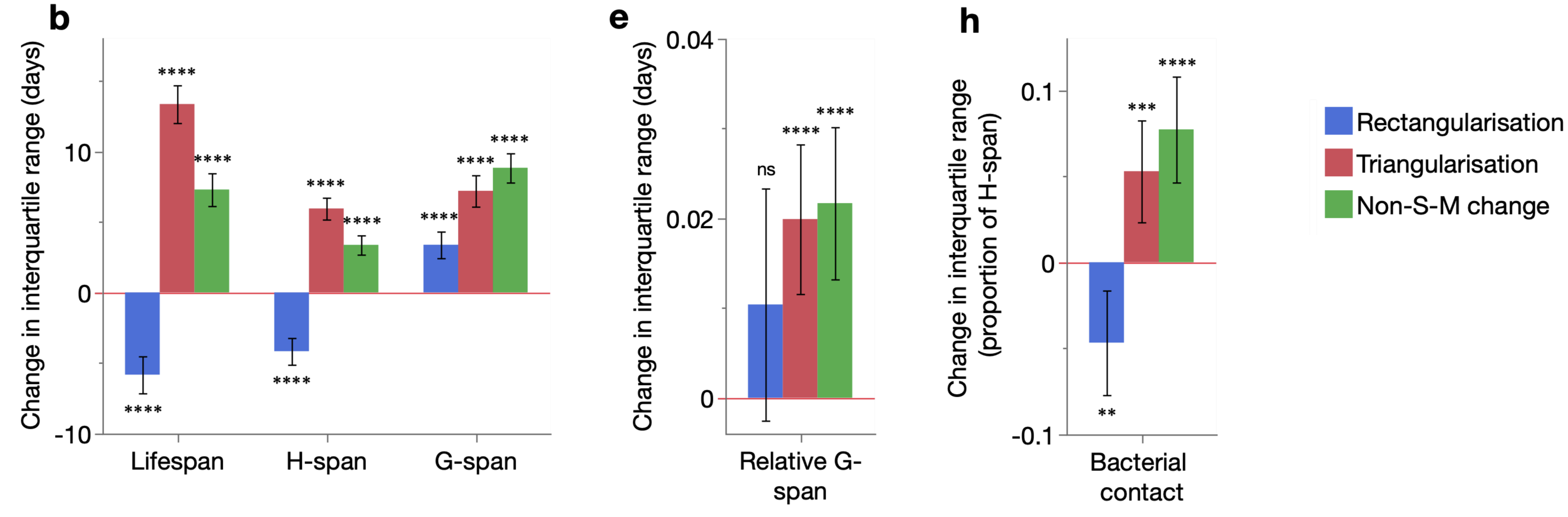
**
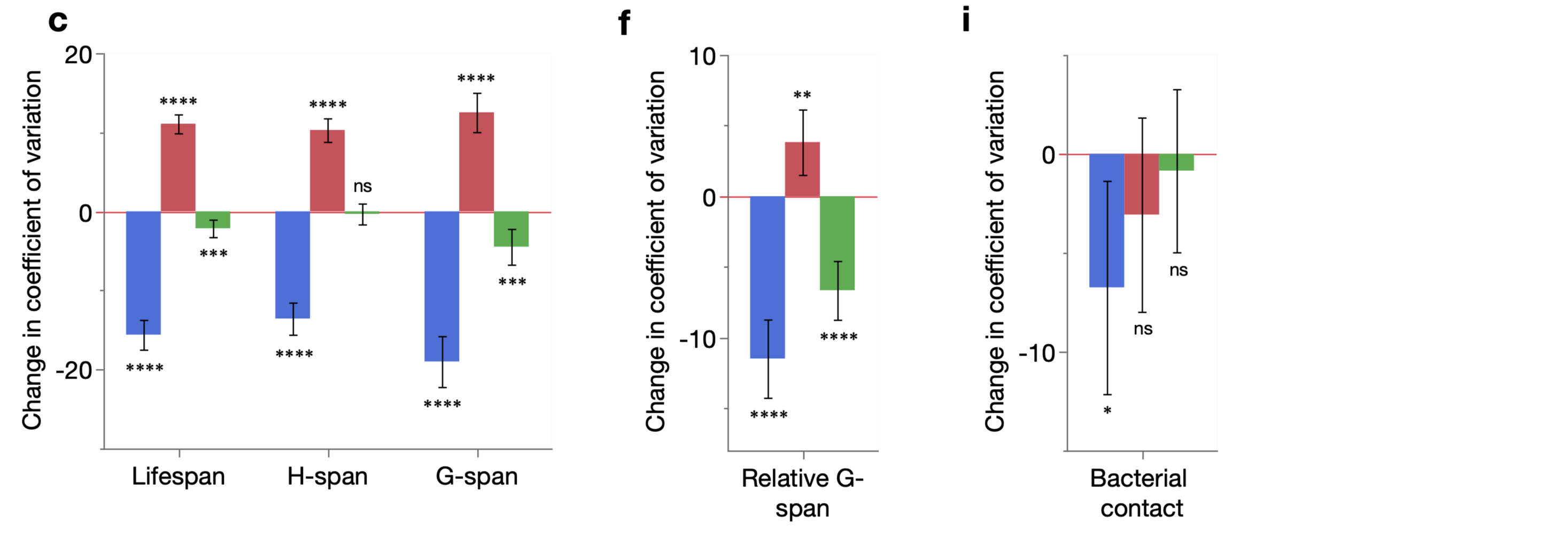


**Extended Data Figure 5. Effects of S-M and non-S-M treatments on variation in the ageing process.** Mean changes in the (**a**, **d**, **g**) median absolute deviation, (**b**, **e**, **h**) interquartile range and (**c**, **f**, **i**) coefficient of variation of (**a**–**c**) lifespan, H-span and G-span, (**d**–**f**) relative G-span and (**g**–**i**) bacterial contact, by S-M^rect^, S-M^tri^ and non-S-M treatments. (**j**) Mean changes in population heterogeneity resulting from changes in subpopulation (P, pIC, pnIC) proportions by S-M^rect^, S-M^tri^ and non-S-M treatments, as measured by Simpson’s Diversity Index. Mean changes in all panels were assessed with two-tailed one sample t-tests (*H*_0_: mean change=0), showing 95% confidence intervals; Benjamini-Hochberg correction of *p* values did not change which treatments reached the 0.05 significance threshold (data not shown). ns *p* > 0.05, * *p* ≤ 0.05, ** *p* ≤ 0.01, *** *p* ≤ 0.001, **** *p* ≤ 0.0001.


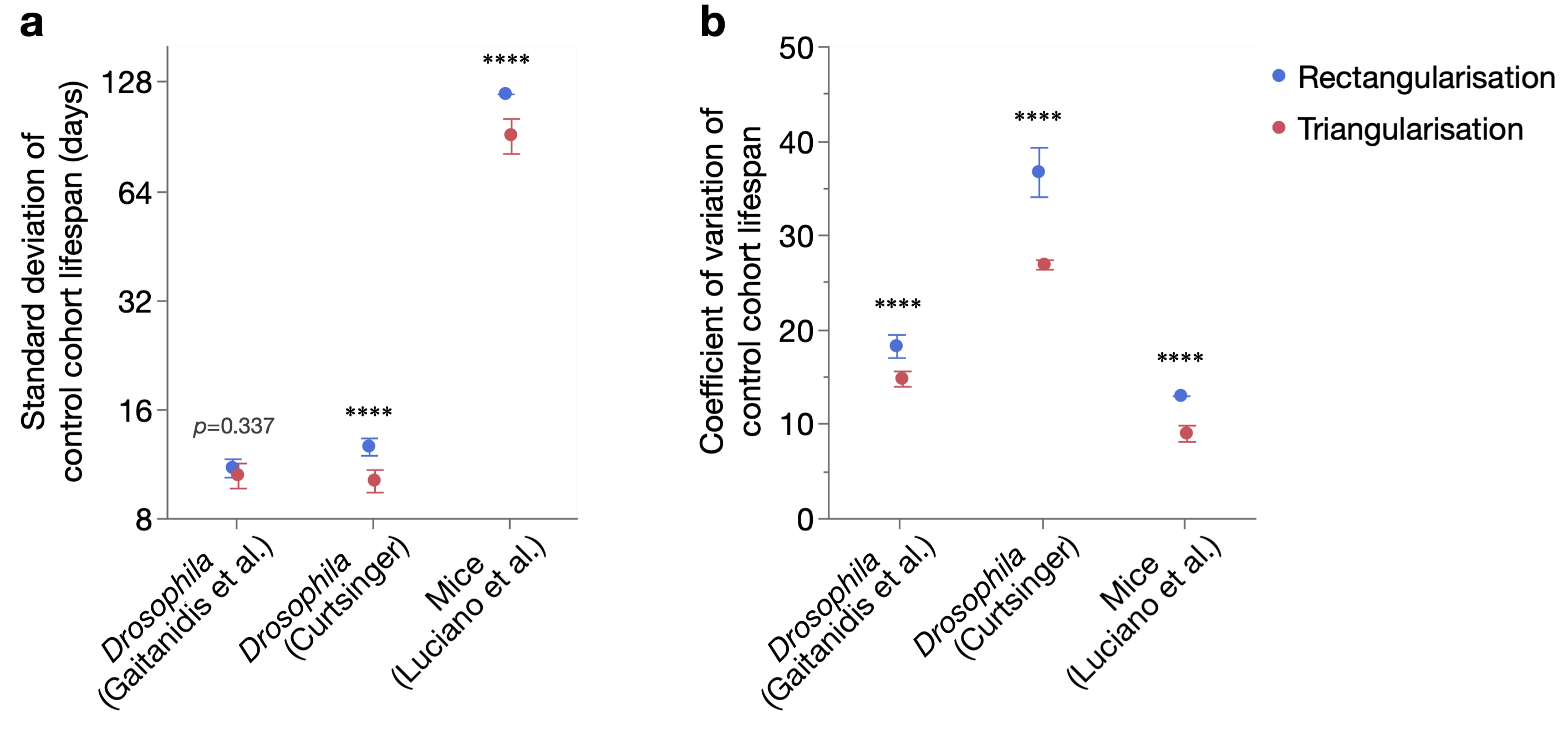


**Extended Data Figure 6. Greater lifespan variation in cohorts undergoing survival curve rectangularisation than triangularisation.** Mean (**a**) standard deviation and (**b**) coefficient of variation of lifespan of control cohorts that underwent S-M^rect^ versus S-M^tri^ treatments (respectively, for Gaitanidis et al., Curtsinger, Luciano et al.: *n*=37, 33; 15, 10; 3, 15). Differences in means were assessed with two-tailed Student’s t-tests, showing 95% confidence intervals. In all panels: ns *p* > 0.05, * *p* ≤ 0.05, ** *p* ≤ 0.01, *** *p* ≤ 0.001, **** *p* ≤ 0.0001.
