# Supplementary material for: The Strehler-Mildvan mortality correlation arises from changes in the variability of ageing
Source: bioRxiv. 2026 Feb 17:2026.02.15.705972. Preprint. [Version 1] doi: 10.64898/2026.02.15.705972 (PMC12934673; doi:10.64898/2026.02.15.705972)
Supplement: Supplement 2 [file media-2.docx]

Supplementary Information

**The Strehler-Mildvan mortality correlation arises from changes in the variability of ageing**

Contents Summary

**Supplementary Table 1.** Lifespan and Gompertz statistics for all cohorts and trials.

**Supplementary Table 2.** Anderson-Darling goodness-of-fit test statistics for all cohorts.

**Supplementary Table 3.** Morbidity-compressing S-M^rect^ treatments.

**Supplementary Table 4.** Morbidity-compressing S-M^tri^ treatments.

**Supplementary Table 5.** Morbidity-compressing non-S-M treatments.

**Supplementary Table 6.** Lifespan and Gompertz statistics for *Drosophila* cohorts in Gaitanidis et al., (2019).

**Supplementary Table 7.** Lifespan and Gompertz statistics for *Drosophila* cohorts in Curtsinger (2015).

**Supplementary Table 8.** Lifespan and Gompertz statistics for mouse cohorts in Luciano et al., (2024).

**Supplementary Table 9.** Lifespan and Gompertz statistics for mouse cohorts in Shahmirzadi et al., (2020).

**Supplementary Table 10.** Lifespan and Gompertz statistics for mouse cohorts in Borland et al., (2024).

**Supplementary Table 1. Lifespan and Gompertz statistics for all cohorts and trials.** N2, wild-type. [C], combined (pooled) data from all trials; carb., carbenicillin. Mean lifespan and its standard errors were obtained by Kaplan-Meier survival analysis in JMP, and Gompertz parameters and their associated statistics (LCI: lower 95% confidence interval; UCI: upper 95% confidence interval) obtained by maximum likelihood estimation in WinModest. In each cohort section, each row below the first corresponds to one trial. Although experiments for the 30 cohorts were not always performed simultaneously due to practical constraints, inter-trial lifespan variation was well controlled (Extended Data Figure 1); such variation was greater in the *daf-2* mutants, a property of these strains noted in previous studies from this laboratory. The analyses in this study utilise the pooled data [C] from all trials, so lifespan and Gompertz statistics are not displayed for individual trials. Two additional larger trials were performed for all N2, *daf-2(m577)*, *daf-2(e1368)* and *daf-2(e1370)* cohorts, whose raw data can be accessed (Zhang and Gems, 2026).

**Supplementary Table 2. Anderson-Darling goodness-of-fit test statistics for all cohorts.** Test statistics and *p* values for parametric bootstrap Anderson-Darling tests, to assess goodness-of-fit of the 30 cohorts to the MLE Gompertz distributions. This test assesses overall, tail-weighted fit (here as the sum of squared, tail-weighted deviations between these functions across all ages). For each cohort, observed A-D statistics were compared against null distributions generated from 5000 parametric bootstrap populations simulated in JMP under the fitted Gompertz distribution of interest (MLE parameters obtained in WinModest), using generating random survival proportions (Uniform(0,1)) and population sizes matching the observed populations (including censored individuals). Monte Carlo *p* values were computed as the proportion of bootstrap A-D statistics greater than or equal to the observed statistic, applying the Phipson–Smyth correction (addition of 1 to numerator and denominator). *p* values less than 0.05 are shaded.

**Supplementary Table 3. Morbidity-compressing S-M^rect^ treatments.** Temperature, antibiotic usage and genotype of S-M^rect^ treatments that decreased relative G-span. Each treatment comprises a pair of cohorts (control and treatment cohorts), and each row contains one treatment (15 in total).

**Supplementary Table 4. Morbidity-compressing S-M^tri^ treatments.** Temperature, antibiotic usage and genotype of S-M^tri^ treatments that decreased relative G-span. Each treatment comprises a pair of cohorts (control and treatment cohorts), and each row contains one treatment (72 in total).

**Supplementary Table 5. Morbidity-compressing non-S-M treatments.** Temperature, antibiotic usage and genotype of non-S-M treatments that decreased relative G-span. Each treatment comprises a pair of cohorts (control and treatment cohorts), and each row contains one treatment (39 in total).

**Supplementary Table 6. Lifespan and Gompertz statistics for *Drosophila* cohorts in Gaitanidis et al., (2019).** Mean lifespan and its standard errors were obtained by Kaplan-Meier survival analysis in JMP, and Gompertz parameters and their associated statistics (LCI: lower 95% confidence interval; UCI: upper 95% confidence interval) obtained by maximum likelihood estimation in WinModest.

**Supplementary Table 7. Lifespan and Gompertz statistics for *Drosophila* cohorts in Curtsinger (2015).** Mean lifespan and its standard errors were obtained by Kaplan-Meier survival analysis in JMP, and Gompertz parameters and their associated statistics (LCI: lower 95% confidence interval; UCI: upper 95% confidence interval) obtained by maximum likelihood estimation in WinModest. *These data were extracted from event history graphs in Curtsinger (2015), producing 100 pseudo-individuals per cohort (see Methods).

**Supplementary Table 8. Lifespan and Gompertz statistics for mouse cohorts in Luciano et al., (2024).** Mean lifespan and its standard errors were obtained by Kaplan-Meier survival analysis in JMP, and Gompertz parameters and their associated statistics (LCI: lower 95% confidence interval; UCI: upper 95% confidence interval) obtained by maximum likelihood estimation in WinModest.

**Supplementary Table 9. Lifespan and Gompertz statistics for mouse cohorts in Shahmirzadi et al., (2020).** Mean lifespan and its standard errors were obtained by Kaplan-Meier survival analysis in JMP, and Gompertz parameters and their associated statistics (LCI: lower 95% confidence interval; UCI: upper 95% confidence interval) obtained by maximum likelihood estimation in WinModest.

**Supplementary Table 10. Lifespan and Gompertz statistics for mouse cohorts in Borland et al., (2024).** Mean lifespan and its standard errors were obtained by Kaplan-Meier survival analysis in JMP, and Gompertz parameters and their associated statistics (LCI: lower 95% confidence interval; UCI: upper 95% confidence interval) obtained by maximum likelihood estimation in WinModest.

**References**

Zhang B & Gems D (2026). Slowed Gompertzian ageing in long-lived *C. elegans* results from expansion of decrepitude, not decelerated ageing. *bioRxiv* https://doi.org/10.1101/2025.04.13.648378
